# Integration of 3D bioprinting and multi-algorithm machine learning identified glioma susceptibilities and microenvironment characteristics

**DOI:** 10.1038/s41421-024-00650-7

**Published:** 2024-04-09

**Authors:** Min Tang, Shan Jiang, Xiaoming Huang, Chunxia Ji, Yexin Gu, Ying Qi, Yi Xiang, Emmie Yao, Nancy Zhang, Emma Berman, Di Yu, Yunjia Qu, Longwei Liu, David Berry, Yu Yao

**Affiliations:** 1 Shanghai University of Traditional Chinese Medicine, Shanghai, China; 2 Department of NanoEngineering, University of California San Diego, La Jolla, CA USA; 3 Department of Statistics, University of California Davis, Davis, CA USA; 4 Department of Neurosurgery, Huashan Hospital, Shanghai Medical College, Fudan University, Shanghai, China; 5 National Center for Neurological Disorders, Shanghai, China; 6 Shanghai Key Laboratory of Brain Function and Restoration and Neural Regeneration, Shanghai, China; 7 Immunology Laboratory, Neurosurgical Institute of Fudan University, Shanghai, China; 8 Shanghai Clinical Medical Center of Neurosurgery, Shanghai, China; 9 Cyberiad Biotechnology Ltd., Shanghai, China; 10 Department of Human Biology, University of California San Diego, La Jolla, CA USA; 11 Department of Bioengineering, University of California Berkeley, Berkeley, CA USA; 12 Department of Bioengineering, University of California San Diego, La Jolla, CA USA; 13 Alfred E. Mann Department of Biomedical Engineering, University of Southern California, Los Angeles, CA USA; 14 Department of Orthopaedic Surgery, University of California San Diego, La Jolla, CA USA

**Keywords:** CNS cancer, Cancer models

## Abstract

Glioma, with its heterogeneous microenvironments and genetic subtypes, presents substantial challenges for treatment prediction and development. We integrated 3D bioprinting and multi-algorithm machine learning as a novel approach to enhance the assessment and understanding of glioma treatment responses and microenvironment characteristics. The bioprinted patient-derived glioma tissues successfully recapitulated molecular properties and drug responses of native tumors. We then developed GlioML, a machine learning workflow incorporating nine distinct algorithms and a weighted ensemble model that generated robust gene expression-based predictors, each reflecting the diverse action mechanisms of various compounds and drugs. The ensemble model superseded the performance of all individual algorithms across diverse in vitro systems, including sphere cultures, complex 3D bioprinted multicellular models, and 3D patient-derived tissues. By integrating bioprinting, the evaluative scope of the treatment expanded to T cell-related therapy and anti-angiogenesis targeted therapy. We identified promising compounds and drugs for glioma treatment and revealed distinct immunosuppressive or angiogenic myeloid-infiltrated tumor microenvironments. These insights pave the way for enhanced therapeutic development for glioma and potentially for other cancers, highlighting the broad application potential of this integrative and translational approach.

## Introduction

Glioma is a complex central nervous system cancer exhibiting significant genetic and phenotypic heterogeneity among patients^1^. Investigations focusing solely on molecular alterations driving neoplastic events or preclinical studies have translated into limited survival advantages in clinical practice for glioblastoma (GBM), the most lethal type of glioma with a five-year survival rate of 6.9%^1^. Methylation of the O-6-methylguanine-DNA methyltransferase (*MGMT*) promoter has been associated with better prognosis in newly diagnosed GBM patients undergoing radiotherapy and temozolomide (TMZ) maintenance treatment^2^. However, mismatch repair defects and hypermutation following the TMZ treatment contribute to drug resistance, and GBM patients often experience high relapse rates and develop chemoresistance^3^. Immunotherapies that demonstrate promising effects in other cancers also show varied efficacies in GBM^4–6^. Despite extensive attempts in glioma investigations and drug development, therapeutic improvement appears stagnant, necessitating a further understanding of the disease and more accurate treatment evaluations to optimize patient survival rates^7^.

Growing evidence highlights that complex tumor microenvironments, including myeloid cells, significantly impact glioma treatment responses^8,9^. Clinical studies have shown that drugs demonstrating promising outcomes in preclinical settings did not yield significant clinical survival benefits, suggesting that current preclinical models are suboptimal^6,10^. Traditional 2D cell culture fails to recapitulate the heterogeneity of cells and the extracellular matrix (ECM) of the tumor microenvironment in vivo. In GBM, tumor-associated macrophages/microglia (TAMs), a highly plastic population of non-neoplastic cells, constitute a substantial part of the tumor mass and are implicated in GBM malignancy and drug resistance^11–13^. TAMs, originating from two distinct myeloid sources, blood-circulating monocytes, and brain-resident microglia, form a pro-tumor stroma for GBM growth and progression^12^. Creating clinically relevant GBM microenvironments requires precise control of cellular compositions and ECM properties, have been achieved with recent advancements in 3D bioprinting techniques and tissue-relevant biomaterials^14,15^. The enabled crosstalk between tumor cells and stromal cells provides valuable insights into tumor cell dependencies and phenotypic features^16^. However, bioprinting has not been exploited to develop patient-derived models using freshly isolated tumor tissues, which might better recapitulate the original tissues.

In addition, the advancement of machine learning has facilitated better pattern recognition from large and complex datasets, such as next-generation sequencing data. Machine learning has been successfully deployed in various disease management processes and to predict cancer drug responses by integrating multi-omics features, such as gene expression^8,17–20^. However, current workflows face several challenges, including potential overfitting issues due to the abundance of omics data and relatively scarce drug response data, emphasizing the critical importance of feature engineering. Furthermore, current machine learning workflows usually rely on a single algorithm for prediction, which is potentially unsuitable for different drug compounds operating through distinct mechanisms.

In this study, we reported the first integration of 3D bioprinting and machine learning to encompass diverse molecular and cellular features to enhance the assessment of glioma treatment responses and microenvironment characteristics. We first leveraged bioprinting to create biomimetic, patient-derived tissues (PDTs) that closely replicate the original tumor’s genomic characteristics, gene expression patterns, and cellular compositions, maintaining high clinical relevance. The bioprinted models generated with recurrent patient tissues exhibited drug resistance to TMZ treatment, which the *MGMT* promoter (pMGMT) methylation assessment failed to indicate. We next developed GlioML, a multi-algorithm machine learning strategy, to generate independent drug predictors with nine stack-one classic single algorithms and one stack-two weighted ensemble model. GlioML produced reliable drug response predictions based on gene expression data, validated using a GBM cell line, bioprinted PDTs, and bioprinted multicellular GBM-myeloid models. GlioML unveiled unique susceptibility patterns of glioma patients to a range of compounds, several of which were confirmed by the bioprinted patient tissues, including lovastatin, dasatinib, and 1S3R-RSL-3 (RSL), each operating through distinct mechanisms. Further, this integration discerned discrete angiogenic or immunosuppressive phenotypic traits in patient-derived models and GBM-myeloid models developed using either microglia or monocytes. Testing of immunotherapy and targeted therapy, in conjunction with cytokine profiling and immunofluorescent staining of 3D bioprinted GBM models, further reinforced GlioML’s discoveries. Our investigation underscores the synergistic capacity of this approach to generate clinically pertinent treatment evaluations for patients and enrich our comprehension of distinct myeloid characteristics in the tumor microenvironment. This integrative strategy holds potential for further refinement and broad application across diverse cancer types, heralding a transformative era in cancer research and treatment.

## Results

### 3D bioprinted PDTs recapitulated genetic features and clinical drug responses of patient tumors

Surgically resected GBM tumor tissues were obtained from glioma patients spanning varied demographics (Supplementary Fig. S1a). PDT cultures were successfully generated for all obtained patient specimens as drug testing models for 22 adult and 1 pediatric high-grade glioma (HGG) patients, encompassing GBM, astrocytoma, oligodendroglioma, and pediatric HGG. Patient tumor tissues were rinsed and dissociated using collagenase to generate single-cell solutions. The bioink comprised of gelatin methacrylate (GelMA) and hyaluronic acid methacrylate (HAMA), has been formulated to mimic the glioma ECM with greater fidelity than Matrigel commonly used in organoid cultures^16^. The prepolymer solution and the single-cell suspension were thoroughly mixed to generate bioink for individual patients and loaded onto the bioprinter, which had 96 independent light sources at the wavelength of 405 nm, to polymerize the bioink and create PDTs.

**Fig. 1:**
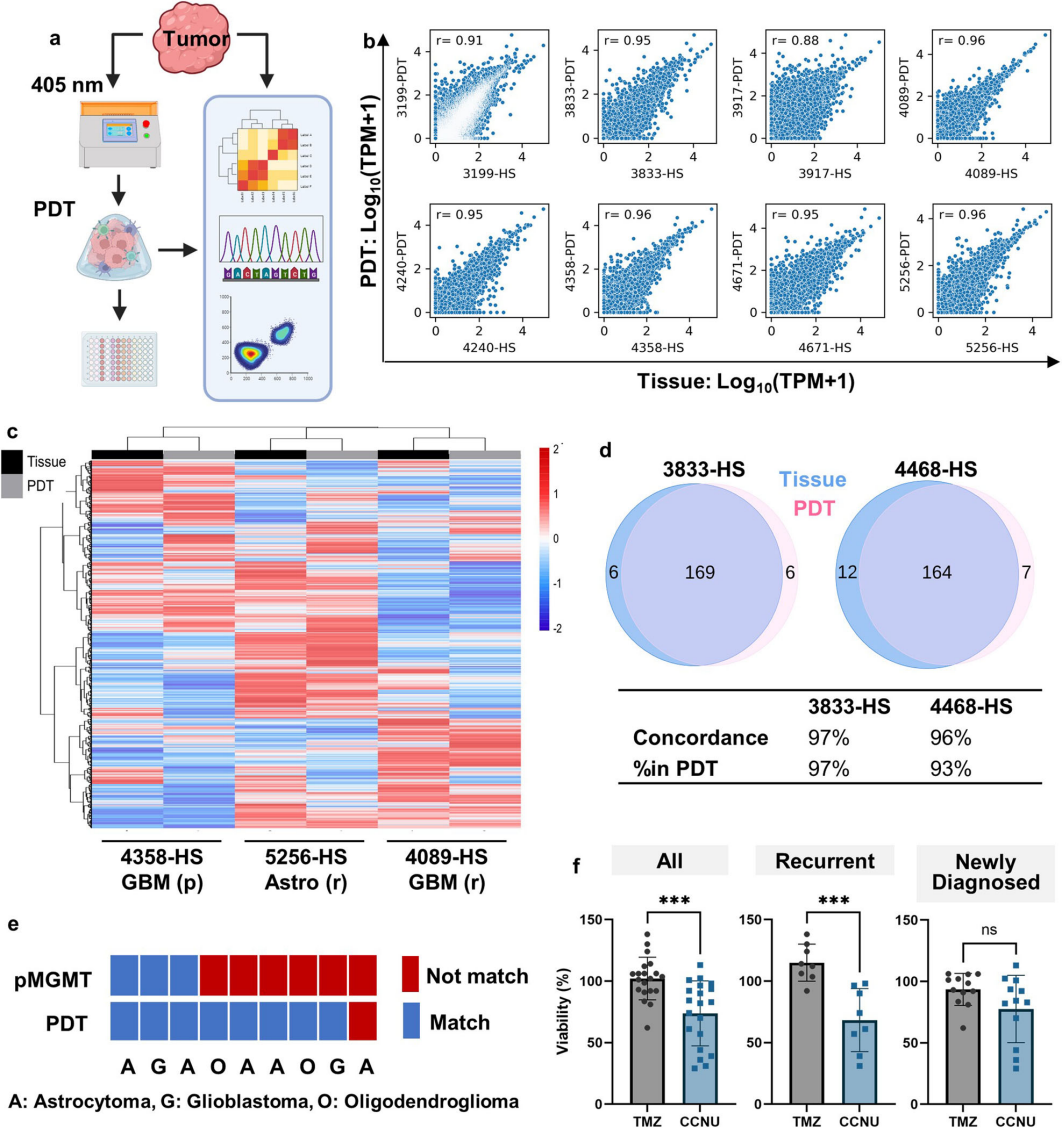
Bioprinting of clinically relevant PDTs for drug evaluations. **a** Schematic of processing workflow for patient specimens, including the generation of PDTs via bioprinting for drug testing and characterization using RNA-seq, WES, and flow cytometry for both primary tissues and PDTs. **b** Pearson correlation graph of the log-transformed gene expression data between primary tissues and PDTs. r: correlation coefficient. **c** Heatmap representation of the transcriptome comparison between three pairs of PDTs and their respective tissue samples, demonstrating high similarity, including a primary GBM patient (4358-HS), a recurrent astrocytoma patient (5256-HS), and a recurrent GBM patient (4089-HS). **d** Venn diagrams illustrating genomic concordance between two patient samples and their PDTs, highlighting over 90% overlap in detected single nucleotide variants. **e** Concordance of the recurrent clinical status to TMZ sensitivity predicted by pMGMT methylation status or assessed through bioprinted PDTs. **f** Comparative efficacy of tumor cell inhibition for TMZ and CCNU in PDTs, showcasing the superior efficacy of CCNU across all patients, especially in recurrent cases.

Pairs of patient specimens and their PDT counterparts were evaluated via RNA sequencing (RNA-seq) and whole exosome sequencing (WES) to determine their molecular statuses (Fig. 1a). RNA-seq showed a high correlation between PDTs and their corresponding patient tissues (Fig. 1b). Gene expression patterns remained mostly consistent between the original tumor and its PDT counterpart, although variations were observed among individuals (Fig. 1c). Principal component analysis (PCA) indicates that most tissue samples and corresponding PDTs cluster closely, except for an oligodendroglioma, a gliosarcoma (GSM), and one GBM specimen showing minor deviations (Supplementary Fig. S1b, c). A comparative analysis of RNA-seq data from patient tissues, bioprinted PDTs, and Matrigel-based patient-derived organoids (PDOs) revealed good correlations between both in vitro models and patient tissues (Supplementary Fig. S2a). The bioprinted models demonstrate a marginally higher correlation coefficient (0.78 and 0.86 for PDT, compared to 0.72 and 0.72 for PDO). This might indicate a better representation of the in vivo condition by the bioprinted PDTs. Despite identical cellular encapsulation, the observed differences may stem from the effects of the different ECM compositions and properties. Furthermore, the majority of mutations identified in patient tissues were mirrored in PDT cultures, confirming the genomic fidelity of the bioprinted PDTs to the original tumors (Fig. 1d). Copy number variations were largely retained across both PDTs and PDOs (Supplementary Fig. S2b). Regarding single nucleotide variants (SNVs) as well as short insertions and deletions (INDELs), both in vitro models successfully conserved the mutational landscape observed in patient tissues (Supplementary Fig. S2c). Nonetheless, PDOs exhibited an increased incidence of de novo mutations, which was suboptimal for replicating native tissue characteristics.

The drug testing involved only first-passage PDTs derived from freshly dissociated cells to maximize clinical relevance. All PDTs were subjected to the current gold standard treatment, TMZ, and lomustine (CCNU), a superior option for recurrent GBM^21^. Additionally, two platinum-based compounds were tested due to their frequent use in pediatric HGG (pHGG) treatment. Viability assessments were conducted post 48 h treatment with 100 μM of the compounds (Supplementary Fig. S3). Lobaplatin was the only drug among the four tested to have both its median and average tumor inhibition rates over 50% in PDT models. PDTs more accurately predicted TMZ resistance in most recurrent adult patients who had already undergone TMZ treatment, compared to the pMGMT methylation status (Fig. 1e). Although pMGMT status (> 10% clinical threshold) indicated TMZ sensitivity in 6 out of 9 adult recurrent patients^22^, drug testing using PDTs revealed that 5 of these 6 patients would not respond to TMZ treatment. This was evidenced by PDTs demonstrating more than 100% cell viability post-treatment (Table 1). The concordance rate of PDTs in reflecting drug resistance among recurrent patients was 89%, in contrast to only 33% for pMGMT status. CCNU exhibited a higher efficacy in inhibiting tumor cells in PDTs, particularly in recurrent GBM patients (Fig. 1f). The median viability of all PDTs after the CCNU treatment was 74%, compared to > 100% for TMZ. Moreover, the median viability of PDTs generated from recurrent patients after the CCNU treatment was 68%, compared to 115% for TMZ (*P* < 0.001). PDT responses to clinical drugs aligned with a meta-analysis identifying CCNU as the most effective chemotherapy for recurrent GBM patients^21^.

**Table 1:** Individual patient clinical information, pMGMT status, and PDT drug responses to clinical drugs TMZ and CCNU.

ID	Age	Sex	Histologic diagnosis	TMZ Treated	pMGMT (%)	TMZ (%)^a^	CCNU (%)^b^
2509-HS	43	M	Astrocytoma, IV, IDH1 mut (R)	Yes	12.0^c^	121	-
2745-HS	51	M	Oligodendroglioma, III, IDH1 mut (R)	Yes	31.2^c^	138	98
2766-HS	41	M	Oligodendroglioma, III, IDH1 mut	No	39.5^c^	106	70
3056-HS	35	F	Astrocytoma, IV, IDH1 mut (R)	Yes	30.7^c^	124	61
3199-HS	27	F	Giant cell glioblastoma, IV, IDH1/2 WT	No	49.0^c^	93	44
3833-HS	21	M	Pediatric High-Grade Glioma, IV, IDH1/2 WT (R)	No	1.7	99	101
3917-HS	43	F	Oligodendroglioma, III (R)	Yes	19.0^c^	114	31
4089-HS	45	M	Glioblastoma, IV, IDH1/2 WT (R)	Yes	38.2^c^	112	57
4240-HS	37	M	Gliosarcoma, IV, IDH1/2 WT (R)	Yes	-	130	39
4358-HS	67	M	Glioblastoma, IV, IDH1/2 WT	No	1.5	81	82
4468-HS	64	M	Glioblastoma, IV, IDH1/2 WT	No	1.5	92	36
4671-HS	55	F	Glioblastoma, IV, IDH1/2 WT	No	7.0	62	29
5256-HS	31	F	Astrocytoma, IV, IDH1 mut (R)	Yes	47.3^c^	92	74
0810-HS	66	M	Glioblastoma, IV, IDH1/2 WT	No	2.0	91	83
1779-HS	52	M	Glioblastoma, IV, IDH1/2 WT	No	2.25	106	92
1869-HS	74	M	Glioblastoma, IV, IDH1/2 WT	No	29.7^c^	-	-
1864-HS	65	M	Glioblastoma, IV, IDH1/2 WT	No	2.75	106	101
10308-HS	39	M	Gliosarcoma, IV, IDH1/2 WT (R)	Yes	1.5	104	90
0911-HS	58	F	Glioblastoma, IV, IDH1/2 WT	No	33^c^	84	113
27691-HS	70	F	Glioblastoma, IV, IDH1/2 WT	No	2.0	102	84
28372-HS	32	F	Astrocytoma, IV, IDH1/2 mut (R)	Yes	3.0	106	96
29259-HS	67	F	Glioblastoma, IV, IDH1/2 WT	No	-	101	94
5371-HS	42	F	Astrocytoma, III, IDH1 mut	No	21.2^c^	99	102

^a^Viability of PDT after TMZ treatment.

^b^Viability of PDT after CCNU treatment.

^c^pMGMT methylation status indicating sensitivity to TMZ treatment.

### Multi-algorithm workflow identified optimal algorithms for diverse compounds

**Fig. 2:**
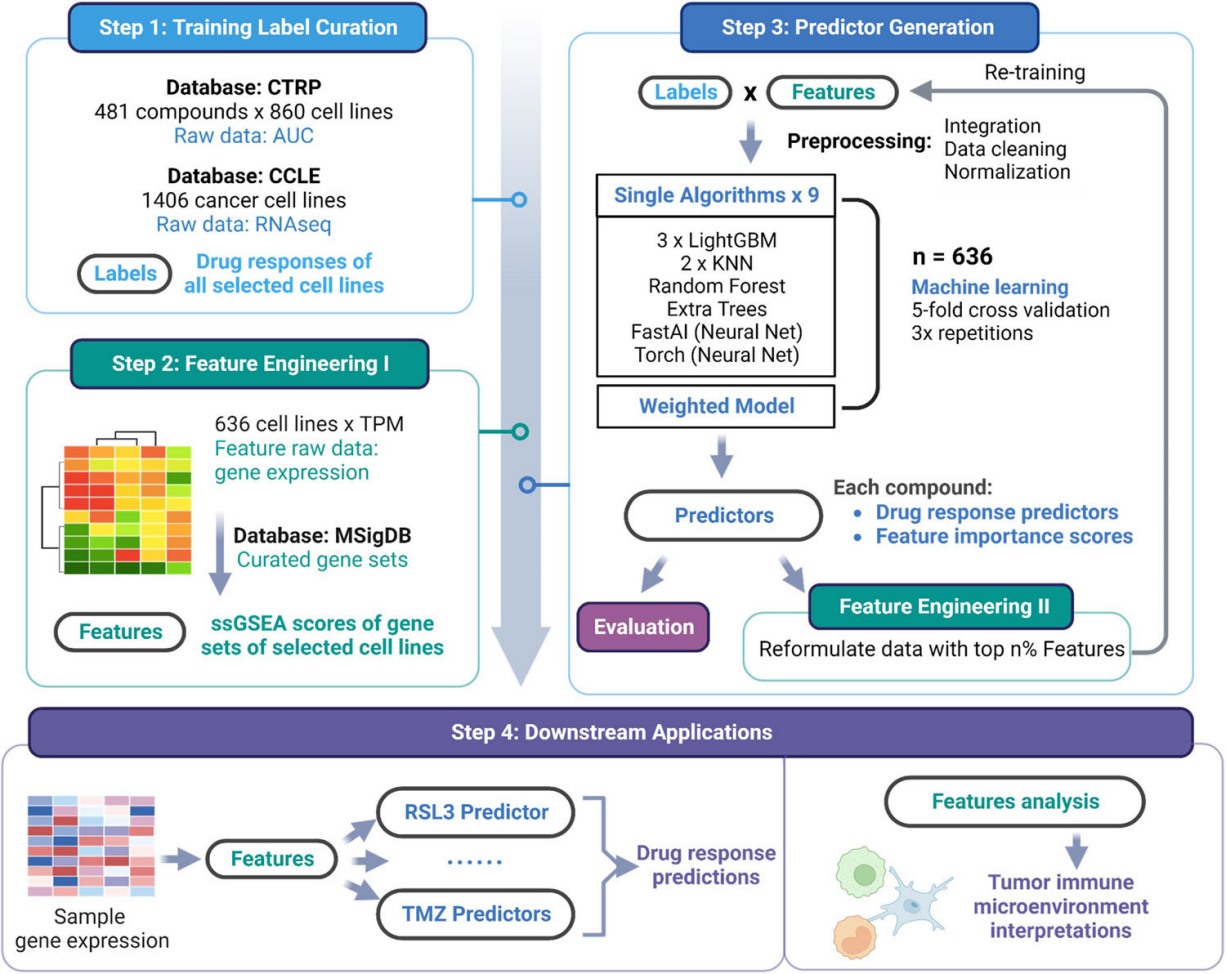
GlioML, a multi-algorithm machine learning approach for drug response prediction. The process consists of four key components: (1) Data Preprocessing — standardization and labeling of the training dataset, (2) Feature Engineering — derivation of relevant features for robust glioma drug response prediction, (3) Training Module — a comprehensive process involving initial training, feature reduction, and performance assessment for individual predictor development, and (4) Downstream Applications — functionalities such as drug response predictions and microenvironment interpretations.

We extracted the gene expression data of established cancer cell lines, as profiled by RNA-seq from the Cancer Cell Line Encyclopedia (CCLE), and the drug response data from the Cancer Therapeutics Response Portal (CTRP), which included 481 drugs and their corresponding drug sensitivity results in well-characterized cancer cell lines as area-under-curve (AUC) values^23,24^. We curated a list of gene sets most relevant to our study, covering canonical pathways, transcription factor-binding sites, and oncogenic signature gene sets, as the initial features for GlioML, the multi-algorithm auto-machine learning workflow^25,26^. Expression data of raw protein-coding genes were converted into single-sample gene set enrichment analysis (ssGSEA) scores of the curated gene sets. These ssGSEA scores provided insight into the level of regulation within each gene set and offered more interpretability than individual gene expression. Of all cell lines in CTRP, 636 were comprehensively characterized in the CCLE and therefore extracted for further use. We employed the AUC values for each cell–compound pairing as labels, while pairs with missing labels were preprocessed and subsequently excluded. We consolidated all this information to generate the raw training data for the workflow (Fig. 2). To mitigate the batch effects in RNA-seq data resulting from disparate sample handling processes, like cell culture methods and library preparations, we performed normalization and data cleansing on the features of both training data and our sample data before submitting them to GlioML (Supplementary Fig. S4).

We incorporated nine supervised base algorithms in our workflow, including three variations of Light Gradient-Boosting Machine (LightGBM), two of K-Nearest Neighbor (KNN), FastAI Neural Network (FastAI), NeuralNetTorch (NNTorch), Random Forest, and Extra Trees, along with a stack-two weighted ensemble model^27–32^. We chose algorithms representing a broad range of learning categories. Each compound underwent independent training to account for its unique mechanisms of action and to accommodate the potential for different algorithms to yield optimal performance. We evaluated model performance via validation scores, with lower absolute values signifying better performance. The tabular data were restructured to incorporate the top n% features, and the models were re-trained to improve performance further.

**Fig. 3:**
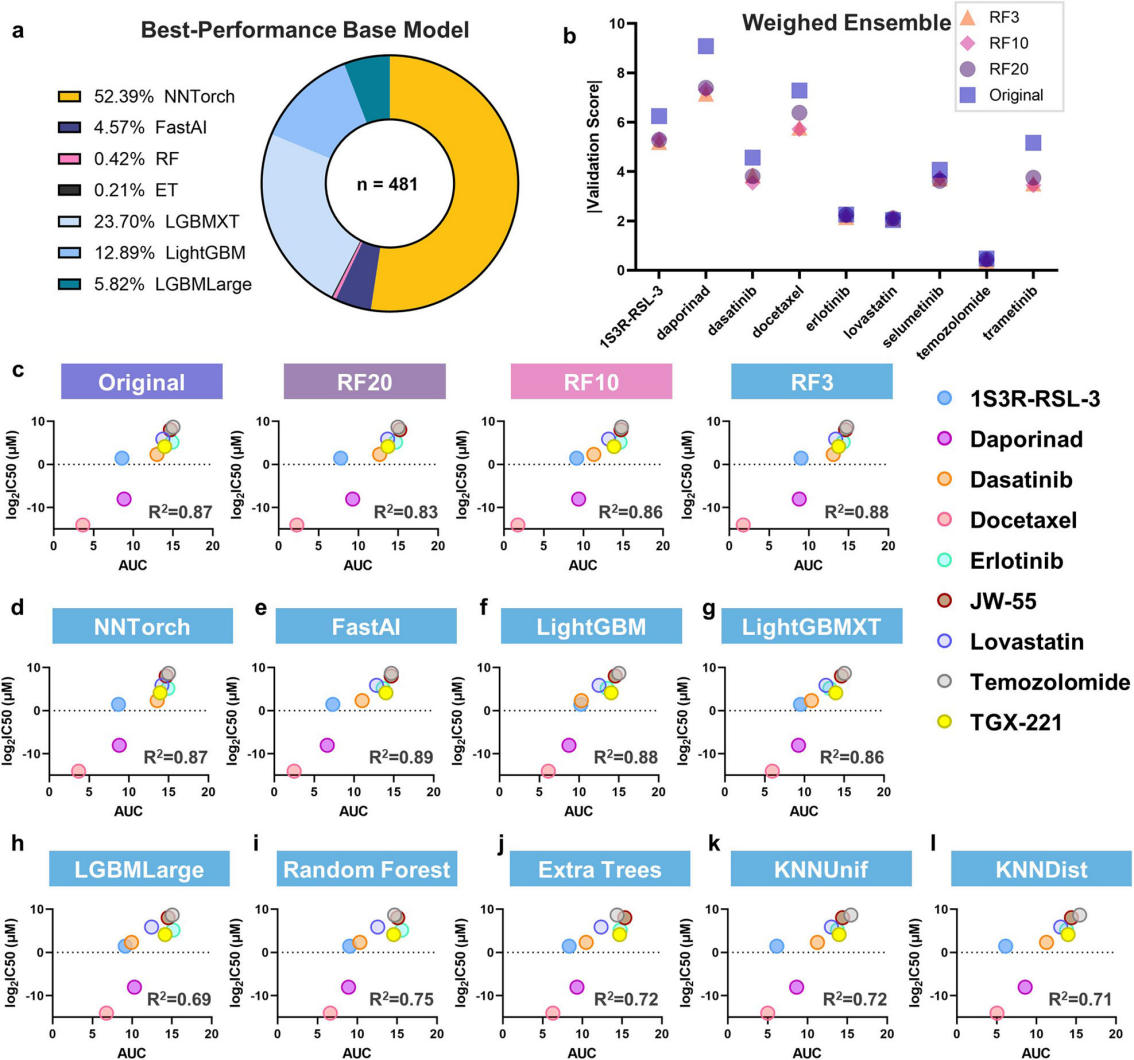
Performance evaluation of GlioML for drug response predictions. **a** The proportion of top-performing predictors generated by different single machine learning algorithms. **b** Absolute validation scores for the weighted ensemble model with feature reduction applied to the top 20%, 10%, and 3% of highest-ranked features. Lower scores imply better performance. **c** Linear regression analysis comparing log-transformed IC_50_ values in GSC with predicted drug response AUC by the weighted ensemble model across all four conditions: original, RF20, RF10, and RF3. **d**–**l** Individual linear regression analyses of log-transformed IC_50_ values and predicted AUC by individual algorithm models: NNTorch (**d**), FastAI (**e**), LightGBM (**f**), LightGBMXT (**g**), LGBMLarge (**h**), Random Forest (**i**), Extra Trees (**j**), KNNUinf (**k**), and KNNDist (**l**). *R*^2^: coefficient of determination.

The training results underscored the superior predictive capacity of neural networks and gradient-boosting algorithms in estimating drug responses from gene expression features. These two categories produced over 98% of the best predictors from single algorithms, while the KNN models did not generate any top predictors (Fig. 3a). Moreover, through an effective combination of base models with optimized weights (Supplementary Fig. S5), the weighted ensemble model outperformed all single algorithms across all compounds in the training datasets (Supplementary Fig. S6).

### GlioML predicted drug response on an independent patient-derived dataset

We next used a line of GBM stem cells (GSCs), CW468, to assess the predictive capacity of the workflow on independently generated data. CW468 was profiled by RNA-seq, and its gene expression data were processed and analyzed. AUC predictions were obtained for all compounds from every algorithm and the weighted ensemble model. The GSCs were then exposed to nine compounds, including TMZ, the standard drug treatment for GBM, with the predicted AUC spanning the entire range. Half maximal inhibitory concentration (IC_50_) values for each drug–compound pair were obtained.

A second round of feature engineering was conducted to prevent potential overfitting due to the original feature-to-sample size ratio, reducing the number of features used in training to a quantity less than the total sample number. The top 20%, 10%, and 3% highest-ranked features were selected for further training, which resulted in approximately 1:1, 2:1, and 6:1 sample-to-feature size ratios, respectively. Reducing the feature size from the original to the top 20% (RF20) significantly enhanced the validation scores for most drugs, except for lovastatin (*P* = 0.03) (Fig. 3b). The validation scores for six of the nine compounds improved when the feature size was reduced to 3% (RF3) compared to 10% (RF10). However, dasatinib, docetaxel, and trametinib experienced diminished performance, suggesting that a 3% cutoff may be excessively stringent for a few compounds. Statistical analysis of validation scores revealed no significant difference between RF20, RF10, and RF3. Thus, decreasing the feature size to a ratio less than 1:1 with the sample size was critical, and additional fine-tuning yielded minor improvements. RF3-trained predictors were utilized for downstream investigations.

A strong linear relationship was observed between the log_2_ transformed IC_50_ values in the GSCs and the predicted AUC from the weighted ensemble model across all four conditions, including the original, RF20, RF10, and RF3. Simple linear regression analyses on the measurements and predictions produced an *R*^2^ value exceeding 0.80 in all groups, with RF3 predictions providing the best fit (Fig. 3c). In the single algorithm models, NNTorch, FastAI, LightGBM and LightGBMXT, exhibited strong linear relationships between the log-transformed IC_50_ values and the predicted AUC (Fig. 3d–g). The other models, including LightGBMLarge (LGBMLarge), Random Forest, Extra Trees, KNN uniform (KNNUnif), and KNN distance (KNNDist), demonstrated less optimal performance on the GSCs (Fig. 3h–l). These results indicated that GlioML could be reliably employed for independent datasets and thus can easily accommodate other cancer types. Furthermore, it was found that the weighted ensemble, neural network, and certain variations of LightGBM models provided the most robust predictions for the evaluated compounds compared to other models, corroborating the training set evaluation.

To further enhance the predictive precision of our multi-algorithm workflow, we employed three strategies: Glioma^+^ (focusing the dataset solely on glioma cases), Metadata^+^ (adding clinical metadata such as age, gender, and pivotal mutation statuses), and Feature^+^ (including a more extensive array of non-linear conditions). Our analysis revealed that the Glioma^+^ approach significantly increased accuracy within the training dataset, as indicated by improved validation scores (Supplementary Fig. S7a). The Glioma^+^ model attained a median validation score of –0.636, markedly better than –1.16 of the base model and –1.14 of the Metadata^+^ and Feature^+^ models. Given the considerable extension in training time and the marginal score enhancement from integrating more metadata or features, coupled with the need for time efficiency, we proceeded with a trial training and evaluation using the Glioma^+^ condition. This strategic narrowing of the dataset also allowed us to incorporate additional base algorithms, specifically CatBoost and XGBoost, additional leading Boosting algorithms other than the LightGBM. The remaining methodology paralleled our preceding multi-algorithmic approach. In the Glioma^+^ workflow, neural networks and gradient-boosting models still emerged as the most effective, yielding ~97% of the top-performing predictors (Supplementary Fig. S7b). The superior performance of these algorithms compared to regression or bagging algorithms can likely be ascribed to their capacity for modeling the intricate patterns present in omic data. Additionally, techniques such as shrinkage and regularization were pivotal in preventing overfitting, a critical concern in datasets where features often outnumber samples, as observed in this study. In contrast, bagging models, including Random Forests, though generally robust, may fall short of fully capturing the nuanced complexity of omics datasets and managing the prevalent noise and outliers. Notably, within the spectrum of boosting algorithms, LightGBM and XGBoost, which utilize asymmetric tree structures, significantly outperformed CatBoost, which employs symmetric trees. When considering the efficiency of neural network models compared to gradient-boosting algorithms, the significantly shorter median fit times for neural network models such as FastAI and NNTorch (5 s and 46 s, respectively) suggest a computational advantage over the boosting algorithms, with CatBoost taking 246 s, LightGBM Large taking 167 s, and XGBoost taking 85 s for median fit time (Supplementary Fig. S7c). This efficiency does not seem to come at the cost of performance, as the neural network models still deliver a comparable number of top-performance predictors.

Nevertheless, upon evaluation with the CW468 dataset, the Glioma^+^ workflow demonstrated reduced performance compared to the original GlioML (Supplementary Fig. S7d). This discrepancy of performance in the training dataset and independent dataset suggested that the improved validation scores may be attributed to overfitting within a constrained training dataset, which in turn could diminish the generalization ability and this was a crucial aspect of the workflow where predictive accuracy across diverse datasets was imperative. Consequently, we proceeded with the original GlioML workflow for further investigation.

### GlioML identified promising compounds and predicted drug efficacies on glioma PDTs

**Fig. 4:**
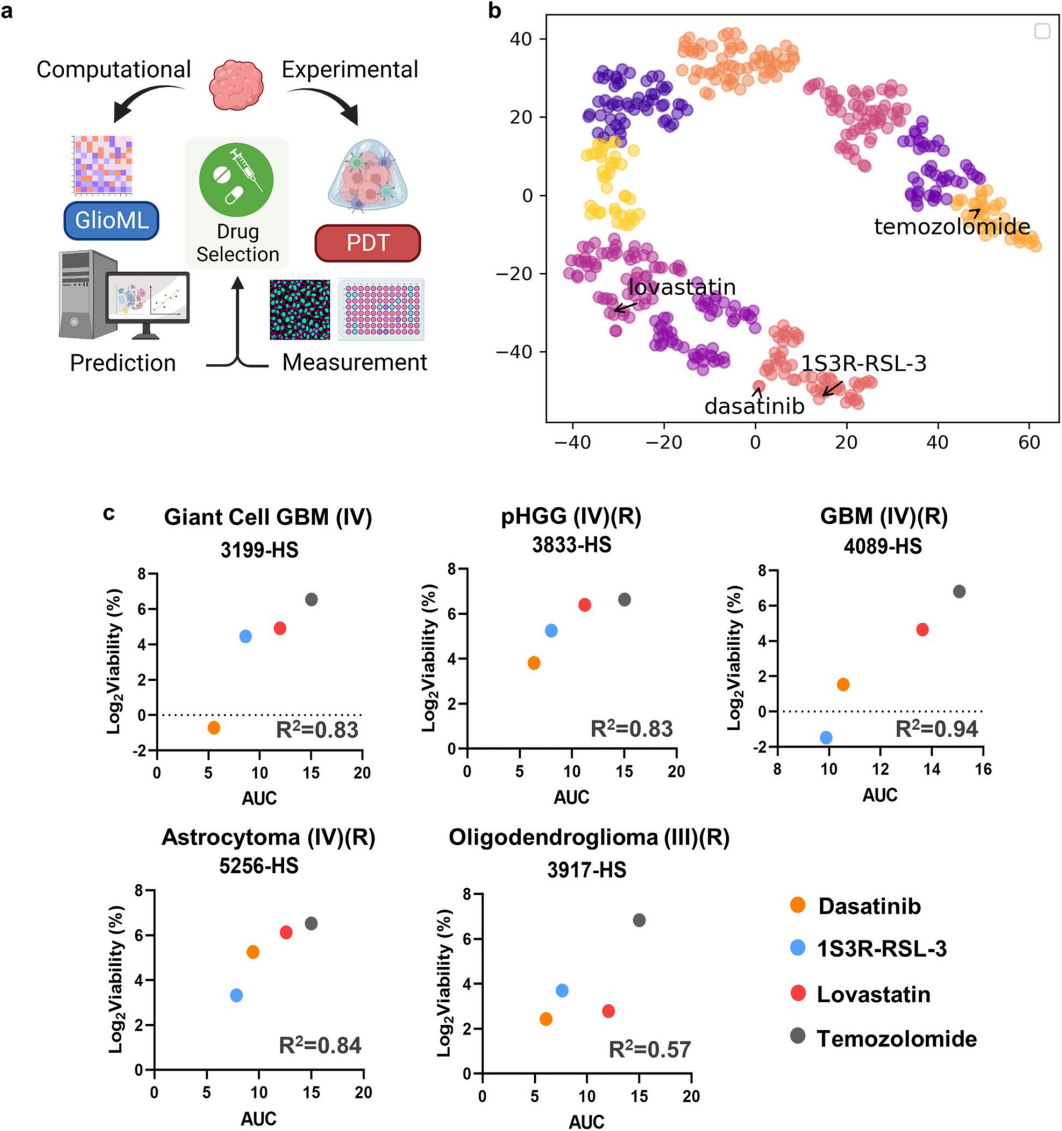
Integration of bioprinted PDTs and GlioML for precision medicine. **a** Schematic of an integrative workflow that combines a computational component (gene expression data acquisition and GlioML prediction) with an experimental part (PDT generation and multiple compound/drug evaluations). **b** t-SNE plot representing all 481 compounds based on their response profiles to patient samples, with RSL and dasatinib in the same cluster, while lovastatin and TMZ belong to different clusters. **c** Linear regression analysis correlating GlioML prediction and log-transformed cell viability in five cases of high-grade gliomas, including three WHO grade IV adult gliomas, one WHO grade III oligodendroglioma, and one pHGG. *R*^2^: coefficient of determination.

We then explored the synergistic potential of integrating 3D bioprinted models and GlioML for predicting and evaluating individual glioma patient drug responses (Fig. 4a). PCA of the AUC matrix for all patients using GlioML effectively distinguished between World Health Organization (WHO) grade III and WHO grade IV gliomas, suggesting that patients at different stages of glioma exhibited different drug response patterns (Supplementary Fig. S8a). Despite the limited number of cells extracted from primary tissues, several PDTs were exposed to three GlioML compounds, along with TMZ. These compounds, including RSL, dasatinib, and lovastatin, demonstrated favorable responses in the CW468. Following treatments with RSL, dasatinib, and lovastatin, the median viabilities were 5.8%, 4.2%, and 50%, respectively. These viabilities significantly surpassed those achieved with TMZ. RSL and dasatinib, lovastatin, and TMZ emerged in three different clusters based on GlioML’s overall predictions for all patient samples (Fig. 4b). Given the effectiveness of RSL and dasatinib, the compounds within this cluster were identified as potential candidates for glioma treatment and could be further explored in the future (Table 2). Although the efficiency of tumor-killing varied among the tested compounds, PDTs treated with CCNU, cisplatin, lobaplatin, dasatinib, lovastatin, and RSL all displayed significantly lower tumor viability than untreated controls (Supplementary Fig. S8b). As PDTs were shown to align with clinical TMZ and CCNU responses, the superior tumor-killing efficacy of GlioML-identified compounds in PDTs supports the potential of GlioML to have a clinical impact on the treatment of glioma.

**Table 2:** Promising compounds for glioma treatment based on GlioML predictions for drug response in patient samples.

Bardoxolone methyl	Gemcitabine	Topotecan	Doxorubicin	Teniposide
Foretinib	NVP-BEZ235	AZD8055	NVP-TAE684	Cucurbitacin I
KPT185	LBH-589	Belinostat	Apicidin	BRD-A86708339
Obatoclax	BRD-K63431240	Pluripotin	Dasatinib	Afatinib
Trametinib	SCH-79797	Methotrexate	Daporinad	CAY10618
PF-3758309	Omacetaxine mepesuccinate	SNS-032	Leptomycin B	Narciclasine
SR-II-138A	CR-1-31B	Dinaciclib	Alvocidib	AZD7762
AT13387	SNX-2112	BI-2536	GSK461364	Triazolothiadiazine
Vincristine	Parbendazole	KX2-391	Paclitaxel	SB-743921
Rigosertib	Docetaxel	Brefeldin A	Ouabain	MLN2238
Avicin D	ML210	ML162	1S3R-RSL-3	Neopeltolide
Oligomycin A				

Subsequently, we evaluated GlioML’s capacity to rank compound efficacy for individual patients by comparing the measured PDT viability to GlioML predictions. GlioML displayed strong predictive potential for various WHO grade IV gliomas, including GBM, giant cell GBM, IDH mutant astrocytoma, and pHGG. However, it demonstrated suboptimal performance for WHO grade III grade oligodendroglioma. Notably, the PDTs treated with all four GlioML compounds, giant cell GBM (3199-HS), recurrent pHGG (3833-HS), recurrent GBM (4089-HS), and recurrent astrocytoma (5256-HS), yielded *R*^2^ values of 0.83, 0.83, 0.94, and 0.84, respectively (Fig. 4c). The oligodendroglioma (3917-HS) yielded a lower *R*^2^ value of 0.57. The median *R*^2^ values were 0.83 for all WHO grade IV gliomas, suggesting that GlioML is particularly adept at interpreting WHO grade IV gliomas.

### Evaluating multimodal therapies through bioprinted PDTs and multicellular models

**Fig. 5:**
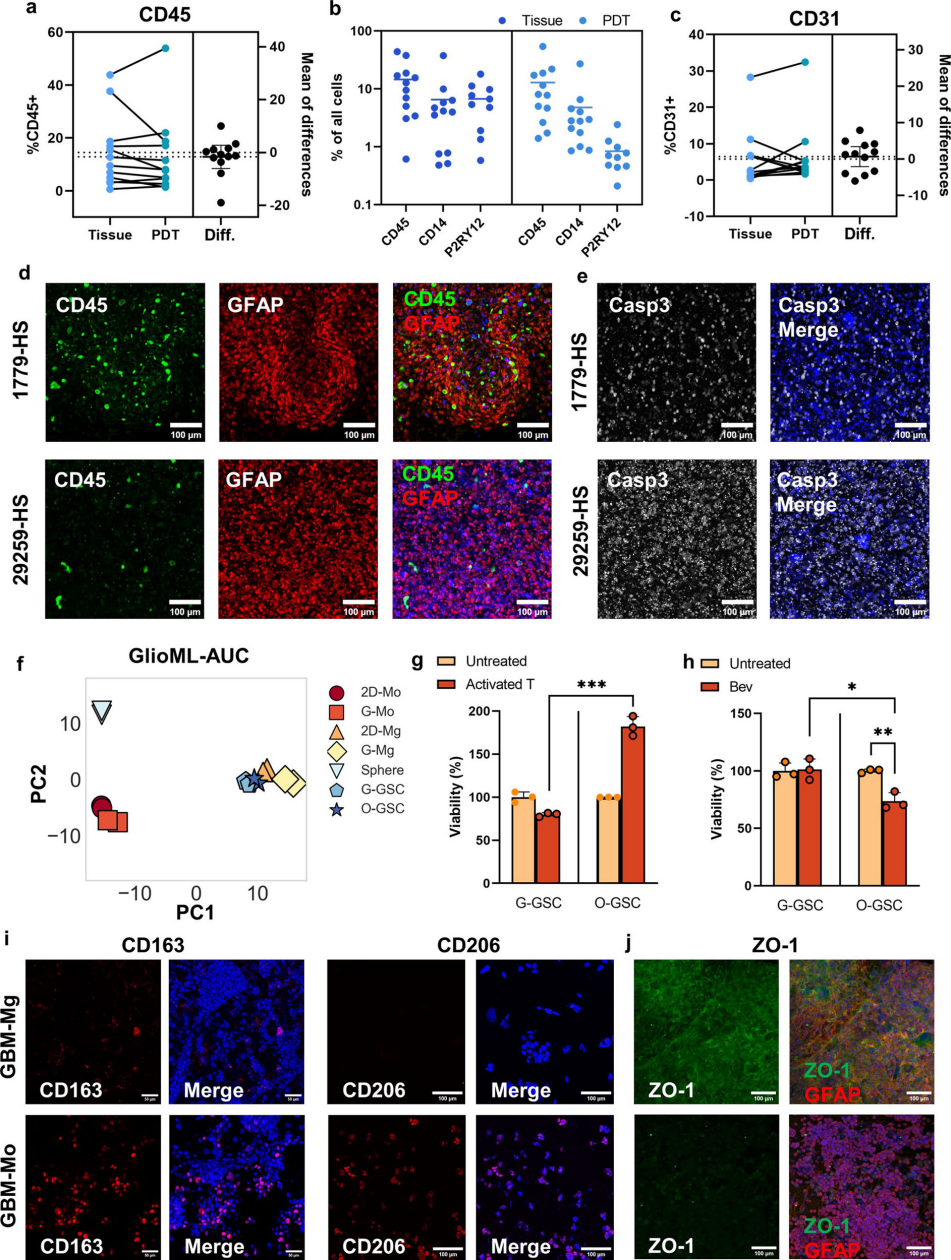
Assessing multimodal treatments in bioprinted PDTs and GBM-myeloid models. **a** Percentage of CD45^+^ cells in patient tissue and bioprinted PDTs. **b** Percentage of CD45^+^, CD14^+^, and P2RY12^+^ cells in patient tissues and PDTs. **c** Percentage of CD31^+^ cells in patient tissue and bioprinted PDTs. **d** Immunofluorescent staining of PDTs derived from 1779-HS and 29259-HS. Green: CD45. Red: GFAP. Scale bars, 100 μm. **e** Immunofluorescent staining of Caspase 3 in PDTs derived from 1779-HS and 29259-HS after T-cell treatment. Scale bars, 100 μm. **f** PCA of drug responses in GSC, Mg, and Mo under different culture conditions. 2D-Mo: 2D cultured monocyte. G-Mo: GSC co-cultured monocyte. 2D-Mg: 2D cultured microglia. G-Mg: GSC co-cultured microglia. Sphere: traditional sphere cultured GSC. G-GSC: microglia-coculturedd GSC. O-GSC: monocyte co-cultured GSC. **g** Cytotoxicity evaluation of T-cell treatment for GSCs in bioprinted GBM-Mg and GBM-Mo models, with E:T ratios of 1:1. **h** Cytotoxicity evaluation of bevacizumab treatment (25 μg/mL) for GSCs in bioprinted GBM-Mg and GBM-Mo models. Immunofluorescence staining of immunosuppressive markers CD163 and CD206 (**i**), and tight junction marker ZO-1 and glial cell marker GFAP (**j**) in GBM-Mg and GBM-Mo models. Scale bars, 100 μm.

HGGs, particularly GBM, exhibit heterogenous non-neoplastic populations, such as immune cells and endothelial cells. Patient samples collected in this study exhibited a varied composition of stromal cells, especially the CD45^+^ immune cells, spanning from < 1% to 44%, and the PDTs effectively preserved the stromal cells (Supplementary Fig. S9). Comparative analysis between 12 matched pairs of patient tissues and PDTs showed no significant variance (*P* = 0.43), barring a few instances where a reduction in CD45^+^ ratio was observed (Fig. 5a). The macrophage populations, including subsets such as CD14^+^ and P2RY12^+^ cells, which are indicative of TAMs of peripheral and cerebral origin, respectively, were found to constitute a considerable fraction of the tumor mass and CD45^+^ cell population, consistent with literature findings. While the CD14^+^ cells were well-maintained, the P2RY12^+^ cells presented challenges in terms of extended in vitro preservation (Fig. 5b). Among the examined patient tissues, CD3^+^ T cells accounted for less than 5% of the cell population in all samples, aligning with the immunosuppressive microenvironment of HGGs. Varying amounts of CD31^+^ endothelial cells were observed in patient samples, spanning from < 1% to 28%. PDTs also successfully preserved the CD31^+^ cells, with no significant differences (*P* = 0.60) observed between 12 pairs of patient tissues and the corresponding PDTs (Fig. 5c).

We further investigated the utility of bioprinted PDTs in assessing the efficacy of activated T cells and Bevacizumab, in addition to the demonstrated capability of evaluating small molecule compounds. We selected PDTs representing two distinct subtypes: CD14-dominant (1779-HS) and P2RY12-dominant (29259-HS). These PDTs were treated with T cells activated by CD3/CD28 microbeads, at an effector:target (E:T) ratio of 1:1. Initially, 1779-HS comprised 41% CD14^+^ cells within 45% of the total CD45^+^ cell population, whereas 29259-HS contained 9% P2RY12^+^ cells within 9% of the total CD45^+^ cells (Fig. 5d). Upon exposure to activated T cells, we observed a higher level of apoptosis, as indicated by Caspase 3 staining (Fig. 5e), in P2RY12-dominant PDTs compared to CD14-dominant PDTs, suggesting greater susceptibility to T-cell mediated cytotoxicity. Differential responses were also noted when PDTs were exposed to Bevacizumab, an anti-angiogenic drug. In CD14-dominant PDTs, post-treatment CD31^+^ cells showed over 50% inhibition relative to the baseline ratio, whereas the untreated control maintained the initial CD31^+^ cell ratio. Conversely, P2RY12-dominant PDTs exhibited a more resistant profile to Bevacizumab, with only a 5% inhibition in CD31^+^ cells post-treatment compared to the baseline. Intriguingly, the untreated P2RY12-dominant PDTs demonstrated a fourfold increase in the CD31^+^ cell ratio, suggesting an environment conducive to angiogenesis.

Despite their significant clinical relevance, PDTs exhibited patient-specific characteristics and presented inconsistent cellular compositions. This included varying quantities of immune cells and cells related to blood vessels, posing challenges for repeatable mechanistic studies. To address this, we employed digital light processing-based bioprinting to create engineered multicellular GBM models to further investigate the differential behaviors observed in CD14-dominant and P2RY12-dominant PDTs. We attempted to reconstruct multicellular models that accurately represented the three cell populations involved in PDT examinations. These included CD14^+^ cells representing peripheral-origin TAMs, P2RY12^+^ cells representing cerebral-origin TAMs, and CD31^+^ cells representing endothelial cells. Accordingly, our assembly included four multicellular combinations: GBM-Monocyte (GBM-Mo), GBM-Microglia (GBM-Mg), GBM-Monocyte-Endothelial Cell (GBM-Mo-EC), and GBM-Microglia-Endothelial Cell (GBM-Mg-EC). These models incorporated tumor cells (CW468), myeloid cells (THP1 monocytes and HMC3 microglia) representing the peripheral and cerebral TAM origins, respectively, and endothelial cells (HUVEC), the critical components of blood vessels. The prepolymer solution used for these models maintained the same concentration and light intensity as that used for the PDTs.

These engineered multicellular models enabled the assessment of various therapeutic approaches, including small molecules identified by GlioML, T-cell therapies, and targeted treatments like Bevacizumab. An initial evaluation of the GlioML’s predictive capacity in these bioprinted multicellular GBM models revealed a linear correlation between the GlioML predictions and the observed drug responses of GSCs in 3D co-cultured models. GlioML demonstrated better predictive performance in GBM-Mg models compared to the GBM-Mo models (Supplementary Fig. S10a, b). The GlioML predictions indicated that traditionally cultured GSCs had lower drug resistance, represented by a smaller AUC value, than 3D myeloid-co-cultured GSCs (Supplementary Fig. S10c). Moreover, we identified similar drug response patterns in microglia-co-cultured GSCs (G-GSCs) and monocyte-co-cultured GSCs (O-GSCs). However, distinct drug susceptibility patterns emerged in GBM-transformed microglia (G-Mg) and GBM-transformed monocytes (G-Mo) (Fig. 5f). G-Mo displayed significantly different drug response patterns than co-cultured GSCs and G-Mg. Given that our bioprinted co-culture models contained a high proportion of myeloid cells, mirroring clinical scenarios where TAMs constitute up to 30% of GBM tissues, the marginally reduced accuracy of GlioML in predicting GSC responses in the GBM-Mo model could likely be ascribed to the GlioML’s analysis focusing primarily on tumor cells.

We subsequently applied the bioprinted GBM-Mg and GBM-Mo models to assess the efficacy of activated T cells and Bevacizumab. Interestingly, although GSCs in the two 3D myeloid-co-culture models showed comparable responses to small molecules, they showed distinct sensitivities to these other treatment modalities. This finding aligned with our observations in the PDTs, where G-GSCs were more susceptible to T-cell therapy than O-GSCs (Fig. 5g). Notably, O-GSCs even demonstrated proliferation under T-cell treatment at an effector:target (E:T) ratio of 1:1. Post-treatment analysis of the GBM-Mo models revealed a reduction in CD8^+^ cytotoxic T cells and an increase in CD4^+^ T cells. Within the CD4^+^ T-cell population, a significantly higher proportion of CD25^+^ T cells was observed in GBM-Mo models compared to GBM-Mg models, suggesting a more immunosuppressive microenvironment in the former. Conversely, with Bevacizumab treatment, O-GSCs demonstrated a higher susceptibility than G-GSCs (Fig. 5h). These results were consistent with those observed in PDTs, suggesting that the differing myeloid cell compositions in these models might foster distinct GBM microenvironments. These environments could be characterized as either immunosuppressive or angiogenic, both of which are recognized as key features of GBM.

### Distinct microenvironment characteristics of bioprinted 3D GBM-myeloid models

To elucidate the factors driving the observed diversity in drug sensitivity, we investigated the bioprinted GBM-myeloid models and GlioML features. We first profiled the gene expression of GSCs under different culture conditions, including traditional sphere culture, bioprinted co-culture (GBM-Mg and GBM-Mo), and bioprinted triculture (consisting of GSCs, microglia, and monocytes together). Traditionally cultured GSCs exhibited significant divergence from all the 3D bioprinted cultures. In contrast, the difference between O-GSC and G-GSC was relatively minor, as indicated by the PCA and differential gene expression analysis (Supplementary Fig. S11a, b). GSC isolated from the triculture model (Tri-GSC) also displayed differences from the co-cultured O-GSC or G-GSC, indicating that combining the two myeloid cell types further impacted the GSCs. We noted distinct GSC morphologies in the GBM-Mg and GBM-Mo models, with G-GSCs appearing more fibroblastic and O-GSCs exhibiting a more rounded shape (Supplementary Fig. S11c). Immunofluorescent staining of hypoxia marker CA9 suggested a more hypoxic state in GSCs co-cultured with myeloid cells than those bioprinted alone (Supplementary Fig. S11d).

Variations in responses to T-cell and Bevacizumab treatments suggested potential roles of immune interactions and angiogenesis. Upon further analysis, GBM-Mo models showed elevated immunosuppressive CD163 and CD206 expression levels (Fig. 5i). According to reverse transcription quantitative polymerase chain reaction (RT-qPCR) data, G-Mo had a substantially higher CD163 expression than its 2D counterparts, with this elevated expression maintained in G-Mo for up to a week. In contrast, G-Mg maintained a low level (Supplementary Fig. S12a). Conversely, G-Mg displayed increased expression of angiogenesis-related VEGFA based on RT-qPCR data (Supplementary Fig. S12b). Immunofluorescent staining revealed a higher ZO-1 tight junction protein expression in GBM-Mg models, while it was undetectable in GBM-Mo models (Fig. 5j). Furthermore, the tube formation assay and endothelial growth evaluation demonstrated that the conditioned medium of GBM-Mg models facilitated more meshes’ formation and increased endothelial cell proliferation (Supplementary Fig. S12c, d).

**Fig. 6:**
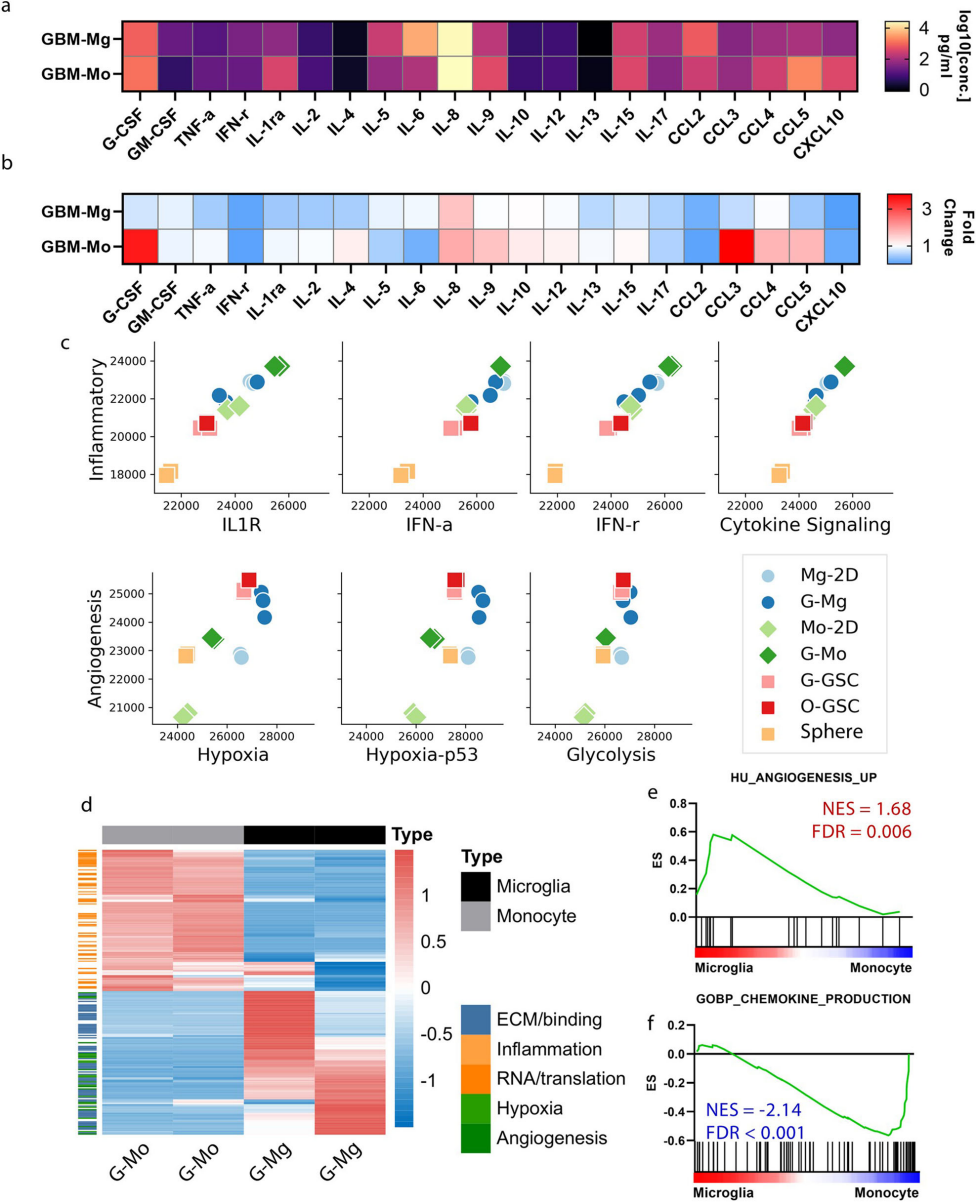
Distinct microenvironment characteristics in GBM-myeloid models. **a** Absolute cytokine abundance in GBM-Mg and GBM-Mo models. **b** Fold-change comparison of cytokine abundance in co-culture supernatants vs supernatants from monocultures of the corresponding cell types. **c** GlioML feature analysis of cells isolated from bioprinted GBM-myeloid models and their traditionally cultured counterparts. **d** Heatmap representation of the top differentially expressed genes in G-Mg and G-Mo, with related pathways annotated on the side. **e** GSEA showing greater enrichment of the angiogenesis pathway in G-Mg. **f** GSEA demonstrating enhanced chemokine production pathway in G-Mo.

Cytokines were pivotal in promoting the immunosuppressive or angiogenic states in the two GBM-myeloid models. In GBM-Mg, the most abundant cytokines were IL8, IL6, and CCL2, while in GBM-Mo, IL8, CCL5, and G-CSF were most prevalent (Fig. 6a). The CCL2/IL6 axis was previously associated with TAM-GBM crosstalk, contributing to enhanced GBM invasiveness^11^. The co-culture supernatant was compared to the mixed supernatant of monoculture of involved cell types (Fig. 6b; Supplementary Fig. S12e). Both 3D co-culture models exhibited reduced levels of pro-inflammatory molecules, such as interferon-gamma (IFN-γ), interferon-gamma-induced protein 10 (CXCL10), and tumor necrosis factor-alpha (TNF-α) compared to the sum of single cultures, indicating the induction of an immunosuppressive state through cellular interactions and transformations in both models, with a more pronounced effect in GBM-Mo (Supplementary Fig. S12f)^33^. Despite the increase in IL8 in both co-culture conditions, other cytokines manifested different alterations in GBM-Mg and GBM-Mo. Most cytokines either did not change significantly or were found at decreased levels in GBM-Mg. Conversely, in GBM-Mo, several chemokines, including G-CSF, CCL3, CCL4, and CCL5, and cytokines, such as IL4, IL9, IL10, IL12, and IL15, were detected at increased levels.

The GlioML feature analysis of cells isolated from the bioprinted models aligned with these observations (Fig. 6c). Cytokine signaling and inflammatory features were the most enriched in the G-Mo, while angiogenic features were dominant in the GBM-Mg microenvironment. Both G-GSC and G-Mg were markedly enriched in angiogenesis-related pathways. Additionally, hypoxia features, hypoxia-induced p53 activation, and glycolysis were most enriched in G-Mg. 3D myeloid-co-cultured GSCs showed an increased expression in these pathways compared to their traditionally cultured counterparts. The GlioML feature analysis aligned with the outcomes of other analysis pipelines, such as gene set enrichment and gene ontology analyses (Fig. 6d–f; Supplementary Fig. S13a–d). We also found these observations consistent across cell lines, as similar gene expression changes were observed in GBM models constructed with another GBM cell line, U251 (data not shown).

## Discussion

We present a pioneering integration of 3D bioprinting and machine learning in a clinically relevant context, advancing experimental and computational methodologies to predict and evaluate multimodal tumor treatment responses. Moreover, this integrative approach enabled the exploration of the intricate tumor-immune microenvironment characteristics.

The bioprinted PDTs using fresh patient samples and a defined hydrogel composition, as the first of their kind in glioma research, represented an excellent alternative to Matrigel-cultured tumor organoids. The cost-effectiveness and batch-to-batch uniformity of the bioink also support the scalability of tissue model production. They faithfully replicated molecular characteristics, cellular composition, ECM compositions, and clinical drug responses found in patient tumors, validating their utility for drug response assessment. Notably, the PDTs demonstrated a capacity to accurately predict TMZ resistance in most recurrent adult patients when pMGMT status failed to predict. This capacity indicates their potential for enhancing treatment planning for GBM patients. Furthermore, these models offer a flexible platform for evaluating patient responses to various therapies, with the ability to differentiate responses to T-cell and anti-angiogenesis targeted therapies, such as Bevacizumab. Despite these advantages, we recognize the limitations of the bioprinted model in forming mature vascular structures within the experimental timeframe, a limitation we aim to address in future studies.

In addition, the GlioML workflow demonstrated proficiency in predicting small molecule efficacy in tumor cells across various in vitro systems, including the bioprinted PDTs developed in the study. Recognizing the distinct mechanistic actions of compounds, we hypothesized that a single algorithm might not generate an optimized predictor for all drugs. The ensemble model of GlioML leveraged the stacking strategy, merging multiple categories of algorithms and validated our hypothesis by surpassing the performance of all individual algorithms. This approach underscored the necessity of context-specific algorithm selection for optimal predictions and bypassed the need for arbitrary model selection and manual parameter tuning typically required in traditional methodologies. The synergy between GlioML and bioprinting yielded a powerful tool for drug assessment and illuminated differential microenvironments within tumor-immune models. This integration identified a cluster of promising yet unexpected candidate compounds. This integrated strategy identified a cohort of unexpected yet promising candidate compounds. RSL3, a ferroptosis activator, demonstrated strong efficacy on GSCs and bioprinted models. Lovastatin, a drug typically used for managing high cholesterol and triglyceride levels, displayed variable efficacy across different PDTs, yet displayed good effectiveness in a few samples. GlioML in combination with 3D bioprinted multicellular models also revealed distinctive drug susceptibility and gene expression patterns in GSCs and myeloid cells. These findings, particularly concerning GBM-transformed microglia and monocytes, enhance our understanding of their unique roles in immunosuppression and angiogenesis. In summary, the versatility of this integrated computational and experimental approach supports its applicability across a range of cancer types, indicating its promising potential for future advancements in personalized cancer therapeutics.

## Materials and methods

### Patient tissue collection

Glioma tissue specimens or blood samples were collected from Huashan Hospital. The research undertaken was approved by the research ethics committee at Huashan Hospital, Fudan University, under the ethics approval numbers: KY2021-670, KY2021-059, KY2023-846. All participating patients provided written consent for using their samples in research endeavors. Pathologists confirmed the classification of the tumor samples. Detailed patient demographic and clinical data are available in Table 1 and Supplementary Fig. S1a.

### Isolation of cells from patient specimens

Surgical samples were cut or minced into 1–3 mm pieces and rinsed thoroughly with Dulbecco’s Phosphate-Buffered Saline (DPBS, Gibco). The mixture was then treated with collagenase type I (Yeasen) and rocked on a shaker at 37 °C for 1 h to aid digestion. The digested samples were rinsed with DPBS. The resulting cell suspensions were collected by centrifugation at 200× *g* for 5 min. A 5-min red cell lysis procedure was performed if excessive red blood cells were present.

### Flow cytometry analysis

Isolated patient cells were resuspended in a DPBS buffer with 40 μg/mL DNase I and analyzed using CytoFLEX flow cytometer (Beckman Coulter). For staining, 1 μL of conjugated CD45 antibody (Biolegend, Cat# 304012) was added to 100 μL cell suspension (0.1 million cells) and incubated for 20 min on ice in the dark. After washing 3 times with DPBS (Gibco), cells were stained with 7-AAD (Biolegend, Cat# 420403) for 10 min at room temperature in the dark. A total of 10,000 events were collected for each sample. Data were analyzed using FlowJo v10.8. All experiments were performed in triplicates.

### GlioML workflow

The GlioML input preparation involved generating features and labels, followed by data cleaning and normalization. Cancer cell line gene expression and therapeutic response AUC data were accessed through the CCLE^23^ and the CTRP^24,34,35^. AUCs were used as labels. Gene-expression TPM values for clinical samples and cell line samples generated in this study were calculated from raw RNA-seq data and transformed to log_2_(TPM + 1) to match the CCLE data format. The ssGSEA module on GenePattern^36^ was used to calculate the ssGSEA scores of each sample using a specific list of gene sets curated from the Molecular Signatures Database (v7.5)^25,37^. The calculated ssGSEA scores for all curated gene sets were used as the feature values. The data cleaning process involved filtering out samples that did not have labels. Normalization was performed by scaling all signature scores to the range of [0, 1].

AutoGluon 0.5.1 was used to build the GlioML workflow. Nine basic models, including FastAI, Neural Network Torch, LightGBM, LightGBM_XT, LightGBM_Large, Random Forest, Extra Trees, KNN_uniform, KNN_distance, and a weighted ensemble model were included. AutoGluon Tabular Predictor was used with specific parameters, including ‘best_quality’ presets, bagging and multi-layer stack ensembling, 3 repeats of 5-fold cross-validation, and random search hyperparameter tuning for 512 times for each model. The problem type was regression; the evaluation metric was mean squared error.

After initial training, feature importance scores were generated using feature_importance(), with subsample_size set to 5000 and num_shuffle_sets set to 10 for highly accurate importance and *P*-value estimates. The scores were presented as a ranking of the most important features to the least important features. These scores were used to interpret the model and identify the most predictive features. Then the top 20, top 10, or top 3 percent of the features were extracted, and the models were re-trained based on the newly formulated training data using the same parameters as the initial training.

### Cell culture

CW468 cells were established by Dr. Jeremy N. Rich lab (UCSD) and cultured in Neurobasal medium (NBM, Gibco, Cat# 21103049) supplemented with 1% B27 minus vitamin A, 1% _L_-glutamine, 1% sodium pyruvate, 1% penicillin/streptomycin (P/S), 10 ng/mL basic human fibroblast growth factor (Peprotech, Cat# AF-100-18B), and 10 ng/mL human epidermal growth factor (Peprotech, Cat# AF-100-15). THP1 cells (American Type Culture Collection, ATCC, Cat# TIB-202) were cultured in Roswell Park Memorial Institute (RPMI) 1640 medium supplemented with 10% FBS and 1% P/S. HMC3 cells (ATCC, Cat# CRL-3304) were cultured in Minimum Essential Medium (MEM, Gibco) medium with 10% fetal bovine serum (FBS), 1% P/S, and 1% non-essential amino acids. HUVEC (Cell Applications, Cat# 200p-05n) were cultured in endothelial cell growth medium v1 (Cell Applications, Cat# 211-500) supplemented with 1% P/S. U251 cells were cultured in DMEM medium with 10% FBS and 1% P/S. All cells were maintained at 37 °C with 5% CO_2_ and passaged with TrypLE (Gibco) or Accutase (Stemcell Technologies) every two to three days. Cell line authentication was conducted for immortalized cell lines, including HMC3 and THP1, using short tandem repeat (STR) profiling and comparison to known reference profiles to ensure data integrity and reliability.

### Drug response evaluation

Drug compounds were pre-dissolved in dimethyl sulfoxide (DMSO) or de-ionized water to a stock solution based on solubility and stored at –20 °C. Cisplatin was obtained from Qilu Pharmaceutical. Lobaplatin was obtained from Hainan Changan International Pharmaceutical. All other tested compounds were obtained from Selleck, including RSL (Cat# S8155), Erlotinib (Cat# S1023), TMZ (Cat# S1237), JW-55 (Cat# S6745), TGX-221 (Cat# 1169), Dasatinib (Cat# S1021), Lovastatin (Cat# S2061), Daporinad (Cat# S2799), and Docetaxel (Cat# S1148).

Before drug treatment, the compounds were diluted in NBM to a concentration that ensured DMSO was less than 0.1% when treating cells. GSCs were bioprinted at 5000 cells per well with 50 μL of medium in 96-well plates and allowed to culture overnight at 37 °C with 5% CO_2_. The next day, 50 μL of 2× drug solution was added to the wells. Cell viability was assessed at the 72-h time point after drug treatment using CellTiter-Glo 3D assay (Promega). Briefly, 100 μL of CellTiter-Glo 3D was added, and the plates were shaken at 300 rpm for 10 min to ensure uniform mixing. The luminescence was measured on a plate reader (Tecan) using luciferase reading. The IC_50_ values were calculated from dose-response curves generated using GraphPad Prism 9 software. All experiments were performed as triplicates.

### Bioprinting PDTs and GBM-myeloid models

PDTs were bioprinted using a light-based bioprinter, Biocube (Cyberiad Biotechnology), with a wavelength of 405 nm, and multicellular GBM-myeloid models were created with a digital light processing bioprinting system, also with a wavelength of 405 nm. Both systems employed matching printing parameters, including a power density (irradiance) of 50 mW/cm^2^ and a scaffold thickness of 0.5 mm. For the PDTs, a cell suspension containing 30 million cells/mL was prepared, with 2 μL of the cell-material suspension required for each sample. For GBM-myeloid cells, a cell suspension composed of equal parts 40 million/mL GBM cells and 20 million/mL THP1 or HMC3 cells was prepared, using 5 μL of cell-material solution for each printed sample.

The prepolymer solution used for all bioprinting processes consisted of 8% GelMA, 2% HAMA (Yuju Technology), and 0.2% lithium phenyl-2,4,6-trimethylbenzoylphosphinate (TCI Chemicals). This solution was thoroughly mixed and stored at 37 °C in the dark before use. The cell suspension was combined with the prepolymer solution at a 1:1 ratio immediately before printing to ensure maximum cell viability. The cell-material mixture was exposed to the bioprinter for an optimized 15–20 s duration. Afterward, the bioprinted constructs were rinsed with DPBS and cultured in a maintenance medium at 37 °C and 5% CO_2_.

The maintenance medium for PDTs consisted of DMEM/F12 supplemented with 1% B27, 1% P/S, 10 ng/mL basic human fibroblast growth factor (Novoprotein), and 10 ng/mL human epidermal growth factor (Novoprotein). The maintenance medium for GBM-myeloid cells contained equal parts CW468 medium and THP1/HMC3 medium. Bioprinted constructs were cultured 7 days before analytical experiments and 3 days before drug treatments. Model monitoring was conducted regularly using optical microscopy.

### Cell isolation from bioprinted samples

The bioprinted samples were incubated with 1 mg/mL collagenase in HBSS (Gibco) for 30–60 min at 37 °C with constant shaking to digest the hydrogel. The samples were then filtered through a 70-μm cell strainer to remove any remaining clumps. The resulting single-cell suspension was collected and centrifuged at 200× *g* for 5 min to collect cells for subsequent analysis.

### WES analysis

Patient samples and their corresponding PDTs and PDOs were subjected to WES analysis and germline corrected using matched blood samples. DNA was extracted using the QIAamp FFPE DNA Tissue Kit (Qiagen). DNA degradation and contamination were assessed on a 1% agarose gel, while concentrations were determined using the Qubit DNA Assay Kit (Life Technologies) and the Qubit 2.0 Fluorometer (Life Technologies). DNA was further fragmented to 150–230 bp, and adapters were ligated at both ends of the fragments. The extracted DNA was then PCR-amplified and hybridized to the KAPA HyperExome Probes (Roche) for enrichment. Libraries were subsequently loaded onto the Illumina NovaSeq 6000 platform (Honsunbio). Patient samples, PDTs, and blood DNA samples were respectively sequenced to average depths of > 400×, > 400×, and > 100× in targeted exonic regions.

The obtained reads were processed using the following pipeline. We aligned the sequencing reads to the human reference genome (hg19) using the Burrows-Wheeler Aligner v0.7.17 and then processed them with Picard v1.119 to remove duplicates. Genome Analysis Toolkit v4.1.9 was used to conduct INDEL realignment and recalibrate base quality scores. Somatic SNVs and INDELs were identified using Mutect2 and Varscan_Indel v2.4.2, respectively, and annotated with ANNOVAR. Copy number variations (CNVs) and gene fusions were detected using CNVkit v0.9.9 and Lumpy v0.2.13, respectively. Purity and ploidy for each sample were manually calculated by comparing the variant allele frequency (VAF) of somatic SNV and CNV log_2_ values of multiple variants within each sample. The final average ploidy/purity was taken from the Sequenza or ABSOLUTE estimate that most closely matched the manual calculation. No precise purity estimates were made for tumor samples with extremely low purity (< 15%).

SNV concordance between tumor–PDT pairs was determined by examining the overlap of variant calls and variant allelic fractions. For each SNV identified in the tumor or PDT, SAMTOOLS Pileup (with a minimum base quality and minimum mapping quality of 10) was run at this position for both samples to compute the variant allele fractions. If read evidence for the SNV was present in both samples (and therefore VAF > 0), the SNV was considered concordant. SNVs called by two or more variant callers in at least one of the samples were included. CNVs were compared between PDTs and tumors by plotting CNV log_2_ values across chromosomes. A threshold of –0.235 and 0.2 was used to delineate the cutoff for deletions and amplifications, respectively, based on a diploid sample with 30% purity. Neutral segments were colored in green, and deletions/amplifications in red. The *y*-axis range was smaller for tumor samples to facilitate CNV identification. SVs were compared between PDTs and tumors by plotting intra- and inter-chromosome rearrangements based on LUMPY results. The SNV landscape was visualized using the Bioconductor package GenVisR v1.8.0. The CNV per gene heatmap was generated using the Bioconductor package ComplexHeatmap v1.17.1.

### Gene expression analysis

Total RNA was extracted from cell pellets using Trizol and Microprep kit (Zymo Research). For RNA-seq, paired-end FASTQ sequencing reads were generated and trimmed using Trim Galore version 0.6.2 and Cutadapt version 2.3, respectively. Transcript quantification was performed using Salmon version 0.13.1 in the quasi-mapping mode using transcripts derived from human Gencode release 30 (GRCh38). Salmon quant files were converted using the R package Tximport, and differential expression analysis was performed using DESeq2. Genes with an adjusted *P*-value (*P*adj) less than 0.05 were considered differentially expressed. To perform gene set enrichment analysis (GSEA), we utilized the GSEA desktop application version 4.1.0 provided by the Broad Institute. The gene sets used for enrichment analysis were obtained from the Molecular Signatures Database (MSigDB). The enrichment analysis was performed using the gene set permutations option, and gene sets with a false discovery rate (FDR) less than 0.25 were considered significantly enriched. The cutoff for gene ontology (GO) analysis was *P*adj < 0.01 and log_2_ fold change > 3. To generate a heatmap, the gene expression data is clustered using a hierarchical clustering approach with the “ward.D2” method and “euclidean” distance, both for rows and columns. Pearson’s correlation coefficient I was computed to measure the linear correlation between each Tissue–PDT pair. Genes that were not detected across a pair of samples (i.e., having zero values) were excluded from each comparison.

For RT-qPCR, cDNA was synthesized from total RNA using the First-Strand cDNA Synthesis Kit (New England Biolabs). After obtaining the cDNA, PowerUp SYBR Green Master Mix (Thermo Fisher Scientific) was used to carry out RT-qPCR analysis with specific primers for *VEGFA* (NM_001025366.3) and *CD163* (NM_203416.3). *VEGFA* Forward: TTGCCTTGCTGCTCTACCTCCA. *VEGFA* Reverse: GATGGCAGTAGCTGCGCTGATA. *CD163* Forward: AAAAAGCCACAACAGGTCGC. *CD163* Reverse: CTTGAGGAAACTGCAAGCCG. For each sample, the RT-qPCR reactions were conducted in triplicate, and the expression levels of the target genes were normalized to *GAPDH* (NM_002046.7) as a reference gene. *GAPDH* Forward: ACAACTTTGGTATCGTGGAAGG. *GAPDH* Reverse: GCCATCACGCCACAGTTTC. The data were analyzed to determine the fold change in G-Mg and G-Mo gene expression at day 7 and day 3 compared to their 2D control. We used a two-tailed *t*-test for statistical analysis, and *P*-values less than 0.05 were considered statistically significant.

### Tube formation assay and endothelial cell growth

A composite medium was prepared using equal parts Neurobasal medium, MEM, and RPMI 1640. For tube formation assessment, conditioned medium from GBM-Mg and GBM-Mo was collected after 72 h of incubation. In each well of an angiogenesis slide (Ibidi), 10 μL of growth factor-reduced Matrigel was added and incubated at 37 °C for 30 min. Subsequently, 50 μL of HUVEC suspension (10,000 cells) was introduced into each well. Cells were cultured in conditioned media from GBM-Mg or GBM-Mo, with non-supplemented endothelial cell growth medium v1 serving as a negative control and endothelial cell growth medium containing 50 ng/mL recombinant VEGF as a positive control. Mesh counts were determined using the ImageJ plugin.

To evaluate endothelial cell growth, 5000 HUVECs were seeded and cultured overnight in endothelial cell growth medium v1. Simultaneously, GBM-Mg and GBM-Mo models were printed. The following day, conditioned medium from GBM-Mg and GBM-Mo was collected and added to the HUVEC culture, with non-supplemented endothelial cell growth medium v1 used as a control. HUVEC medium was replaced every other day using collected conditioned medium. HUVEC growth was assessed on days 1, 3, and 5 post-incubation in the conditioned medium using CellTiter-Glo (Promega), with the experiment conducted in triplicate.

### Isolation, culture, and activation of primary human T cells

Human peripheral blood mononuclear cells (PBMCs) were isolated from diluted whole blood (1:1 in DPBS) (San Diego Blood Bank) using density gradient centrifugation with lymphocyte separation medium (Corning) or purchased from Hycells Biotechnology. Primary human T cells were then isolated from PBMCs using a Pan T-Cell Isolation Kit (Miltenyi) following the manufacturer’s instructions using magnetic-activated cell sorting (MACS) columns. Isolated primary human T cells were cultured with RPMI 1640 with 10% FBS, 1% P/S, and 100 U/mL recombinant human IL-2 (PeproTech). Primary human T cells were activated with Dynabeads Human T-Expander CD3/CD28 (Gibco) for 72 h before usage. Cells were cultured at 37 °C with 5% CO_2_ in a humidified incubator.

### Activated T cell or bevacizumab cytotoxicity assay

Bioprinted PDTs or GBM-myeloid models were fabricated as described earlier. For the multicellular models, luciferase-labeled GSCs were used. For the activated T-cell assay, the bioprinted PDTs or GBM-myeloid models were incubated with activated T cells at an effector-to-target (E:T) ratio of 1:1. The T cells were added to the bioprinted PDTs or models and incubated at 37 °C with 5% CO_2_ for 3 days. The control group contained the bioprinted model without T cells. The assay was performed in triplicate for all conditions.

For bevacizumab evaluation, HUVEC was mixed with the cell mixture at 20 million/mL for printing of the multicellular models. The other printing parameters were the same as previously described. Bevacizumab was tested at a concentration of 25 μg/mL. The bioprinted models containing both HUVEC and the cell mixture, or the bioprinted PDTs, were treated with Bevacizumab for three days. The control group contained the bioprinted model without bevacizumab treatment. The assay was performed in triplicate for all conditions.

The impact of T cells or Bevacizumab on the PDTs was assessed through immunofluorescent staining for Caspase 3, and flow cytometry to quantify CD31^+^ cell populations. The cytotoxicity of T cells or bevacizumab to GSCs in the multicellular models was measured using a luciferase assay system (Promega). The bioprinted models were digested and fully lysed, and the substrate was mixed with cell lysates. Luminescence was measured using a luminometer (Tecan). The assay was performed in triplicate for all conditions.

### Immunofluorescent staining

For immunofluorescent staining, the models were fixed with 1 mL of 4% paraformaldehyde in PBS overnight at 4 °C. Following PBS washing, the cells were permeabilized with 0.1% Triton X-100 for 20 min and subsequently blocked with 5% BSA for 90 min. Primary antibodies against CD163 (1:100 dilution, Proteintech, Cat# 16646-1-AP), CD206 (1:100 dilution, Abcam, Cat# 91992 S), ZO-1 (1:100 dilution, Invitrogen, Cat# 339100), Caspase 3 (1:100 dilution, Proteintech, Cat# 66470-2-lg), CD45 (1:100 dilution, Proteintech, Cat# 65109-1-lg), or GFAP (1:100 dilution, Sigma, Cat# G3893) were diluted in staining buffer (BioLegend, Cat# 420201) and incubated at 4 °C overnight. Samples were rinsed again with PBS for 3 times and then incubated with secondary antibodies (1:200) conjugated with fluorophores for 2 h at room temperature. DAPI (1:1000) was used for nuclear staining. Samples were imaged using a confocal microscope (Leica SP8), and image analysis was performed using ImageJ software. A minimum of three samples were analyzed per condition.

### Luminex for cytokine analysis

Culture supernatants were collected from 3D bioprinted models on day 7. The supernatants were centrifuged at 3000 rpm for 5 min to remove cellular debris. The assay was performed using the Human 27 Cytokine/Chemokine Panel. The samples were read using a Luminex 200 instrument (Luminex Corporation), and the results were analyzed using Milliplex Analyst 5.1 software. Replicates were measured for each condition.

### Statistical analysis

Statistical analysis was performed using GraphPad Prism 9. The data presented are the mean ± SD from a minimum of triplicates. We used a two-tailed *t*-test to analyze the differences between two groups, and for multiple group comparisons, we employed one-way ANOVA followed by Tukey’s post hoc test. We considered a *P*-value below 0.05 to be statistically significant. To assess the relationships between GlioML predictions and measured drug responses, we fit and calculated the coefficient of determination (*R*^2^) to determine the amount of variance explained by the models. To perform dimensionality reduction and visualize complex datasets, we utilized the scikit-learn library v1.1.0 in Python to conduct PCA and t-distributed stochastic neighbor embedding (t-SNE).

### Supplementary information

Suprementary Information

## Data Availability

All raw sequencing data has been deposited in the Sequence Read Archive (BioProject accession number: PRJNA952850) and National Omics Data Encyclopedia (project number: OEP004093). All other data can be obtained upon request by contacting the corresponding authors. All biological materials employed in this manuscript will be provided upon request.

## Material availability

All biological materials employed in this manuscript will be provided upon request.

## Code availability

All computational algorithms used in the manuscript are cited in the respective figure legends and detailed in the Materials and methods section. Further information can be provided upon request.
