# Supplementary material for: Integration of 3D bioprinting and multi-algorithm machine learning identified glioma susceptibilities and microenvironment characteristics
Source: Cell Discov. 2024 Apr 9;10:39. doi: 10.1038/s41421-024-00650-7 (PMC11003988; doi:10.1038/s41421-024-00650-7)
Supplement: Supplementary file 1 — Suprementary Information [file 41421_2024_650_MOESM1_ESM.pdf]

a

**Characteristics**	**All patients (n = 23)**	**Female (n = 10)**	**Male (n = 13)**
**Age group, yr**			
65+	6 (26.1%)	2 (20%)	4 (30.8%)
40 – 64	10 (43.5%)	4 (40%)	6 (46.1%)
20 – 39	7 (30.4%)	4 (40%)	3 (23.1%)
**Histology Grade**			
WHO IV	19 (82.6%)	8 (80%)	11 (84.6%)
WHO III	4 (17.4%)	2 (20%)	2 (15.4%)
**Primary or Recurrent**			
Recurrent	10 (43.5%)	4 (40%)	6 (46.2%)
Primary	13 (56.5%)	6 (60%)	7 (53.8%)

b

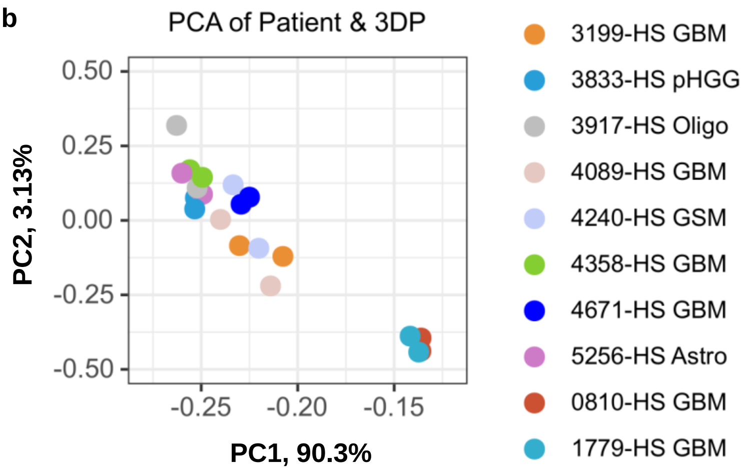

c

	**PC1**	**PC2**
3199-HS	-0.230	-0.085
3199-3DP	-0.208	-0.121
3833-HS	-0.253	0.075
3833-3DP	-0.253	0.038
3917-HS	-0.263	0.319
3917-3DP	-0.252	0.109
4089-HS	-0.240	0.003
4089-3DP	-0.214	-0.220
4240-HS	-0.220	-0.093
4240-3DP	-0.233	0.120
4358-HS	-0.256	0.170
4358-3DP	-0.249	0.145
4671-HS	-0.229	0.054
4671-3DP	-0.225	0.078
5256-HS	-0.249	0.087
5256-3DP	-0.260	0.159
0810-HS	-0.136	-0.395
0810-3DP	-0.136	-0.439
1779-HS	-0.142	-0.388
1779-3DP	-0.137	-0.442

Supplementary Fig. S1. (a) Table of patient demographics of samples collected in this study. (b) Principal component analysis of 10 pairs of patient samples and corresponding PDTs. Each color have two dots, representing the patient sample and its corresponding PDT. (c) PC1 and PC2 values of the principal component analysis for the 10 pairs of tissue samples and bioprinted PDTs.

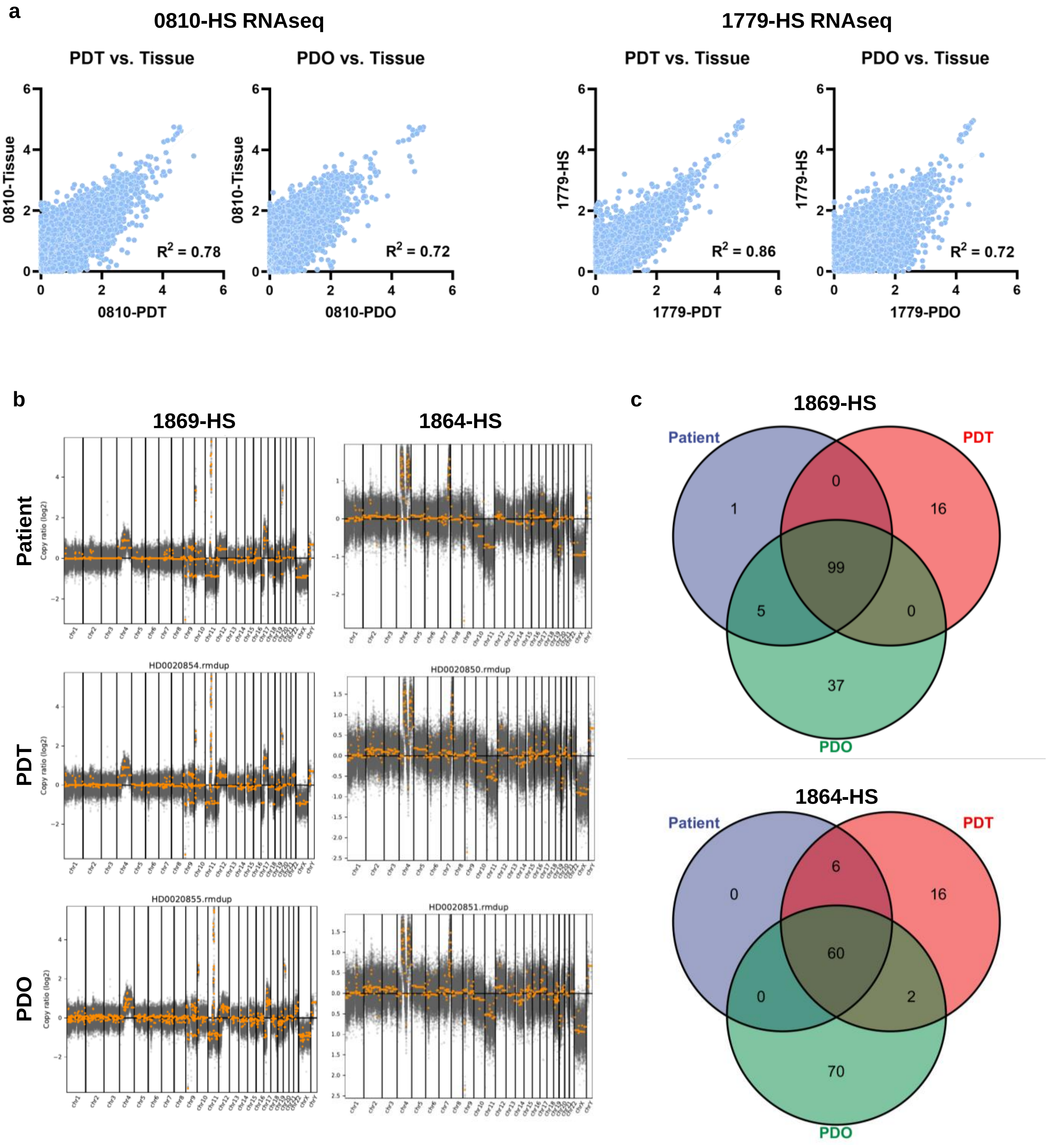

Supplementary Fig. S2. (a) Pearson correlation graph of the Log transformed gene expression data between primary tissues and PDTs/PDOs. (b) Copy number variation of patient sample and corresponding PDT/PDOs. (c) Venn diagrams illustrating genomic concordance between two patient samples and corresponding PDTs/PDOs.

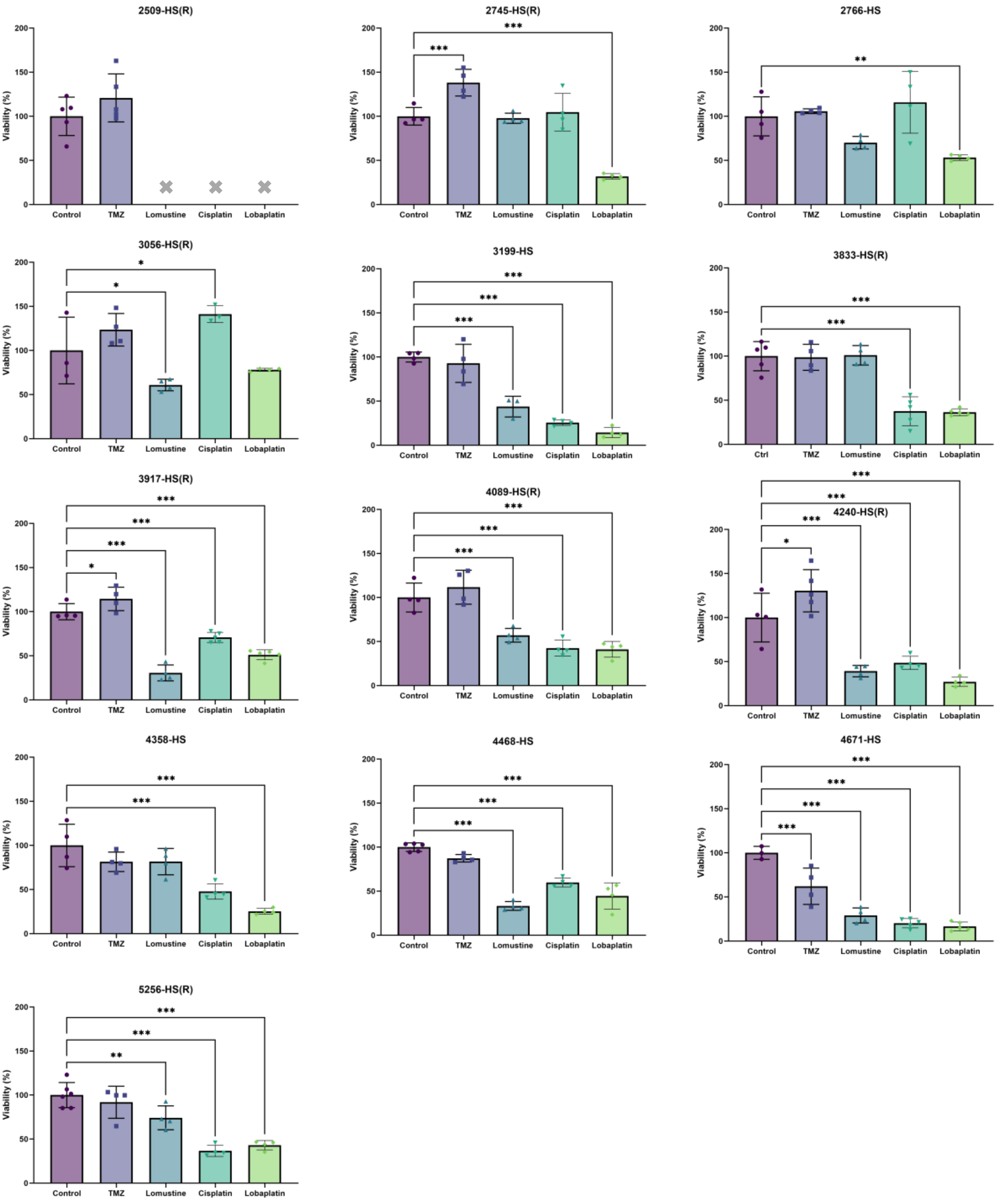

Supplementary Fig. S3. PDT drug responses to clinical drugs, including TMZ, CCNU, Cisplatin, and Lobaplatin, of 13 tested patients. Patient 2509-HS was only tested with TMZ. All other patients were tested with all four compounds. At least three replicates was measured and analyzed. One-way ANOVA was used for statistical analysis. * $P < 0.05$. ** $P < 0.01$. *** $P < 0.001$.

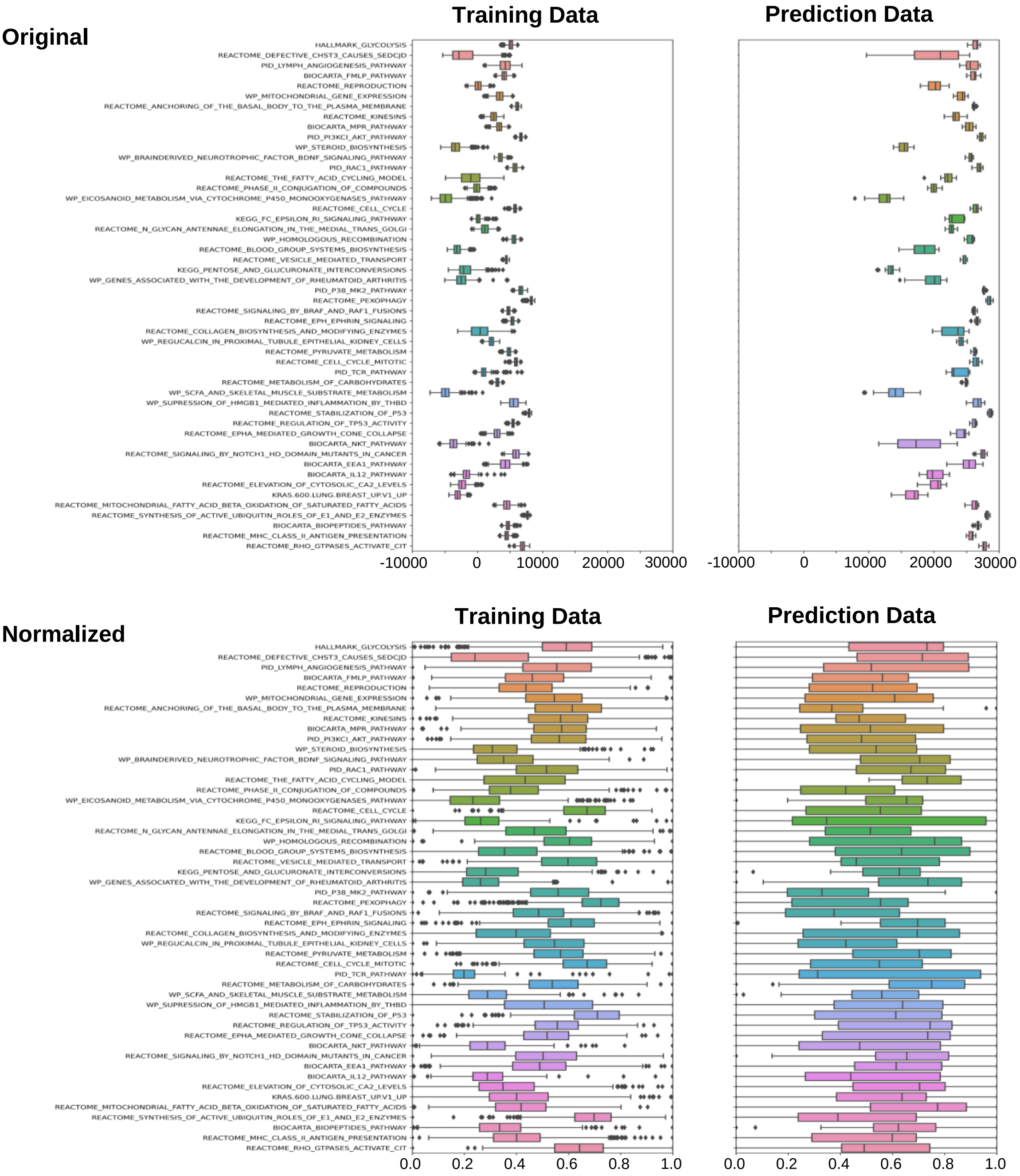

Supplementary Fig. S4. Feature values of training data set and prediction data set before and after normalization.

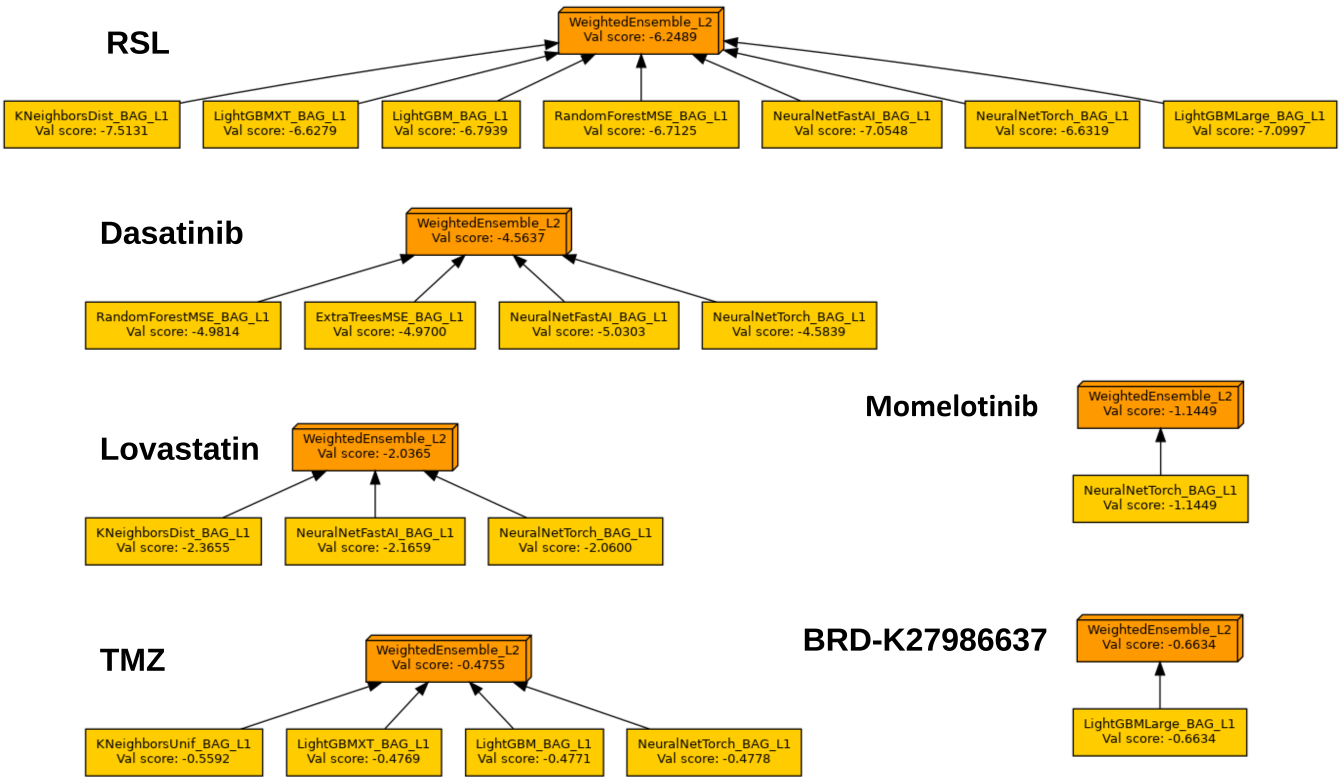

Supplementary Fig. S5. Structure of stack-two weighted ensemble model of tested compounds, including RSL, dasatinib, lovastatin, TMZ, as well as two compounds that only have one contributing stack-one model, including momelotinib and BRD-K27986637.

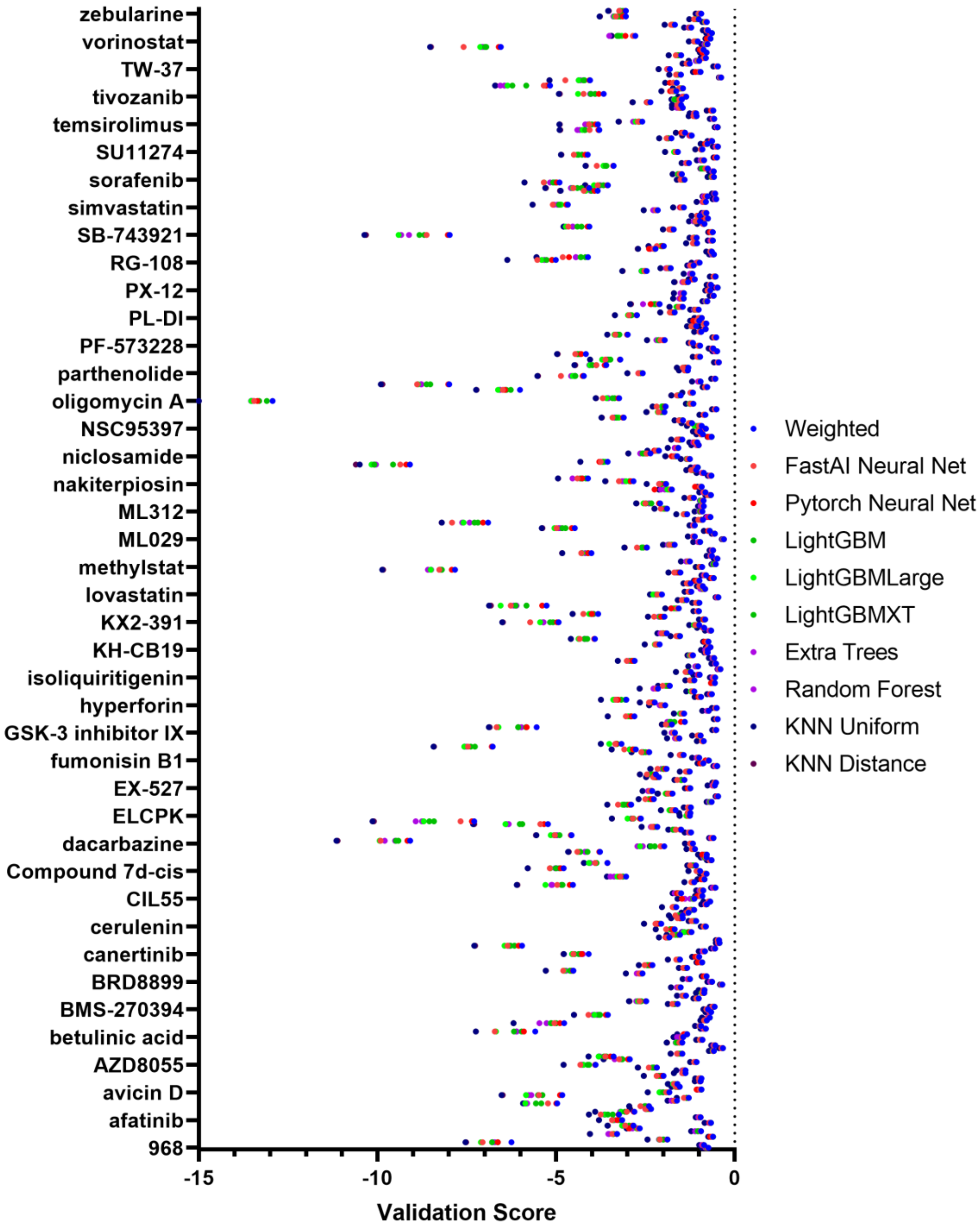

Supplementary Fig. S6. Validation score of all 481 compounds of the stack-two weighted model and 9 stack-one algorithms. Larger (less negative) value indicate better performance after training.

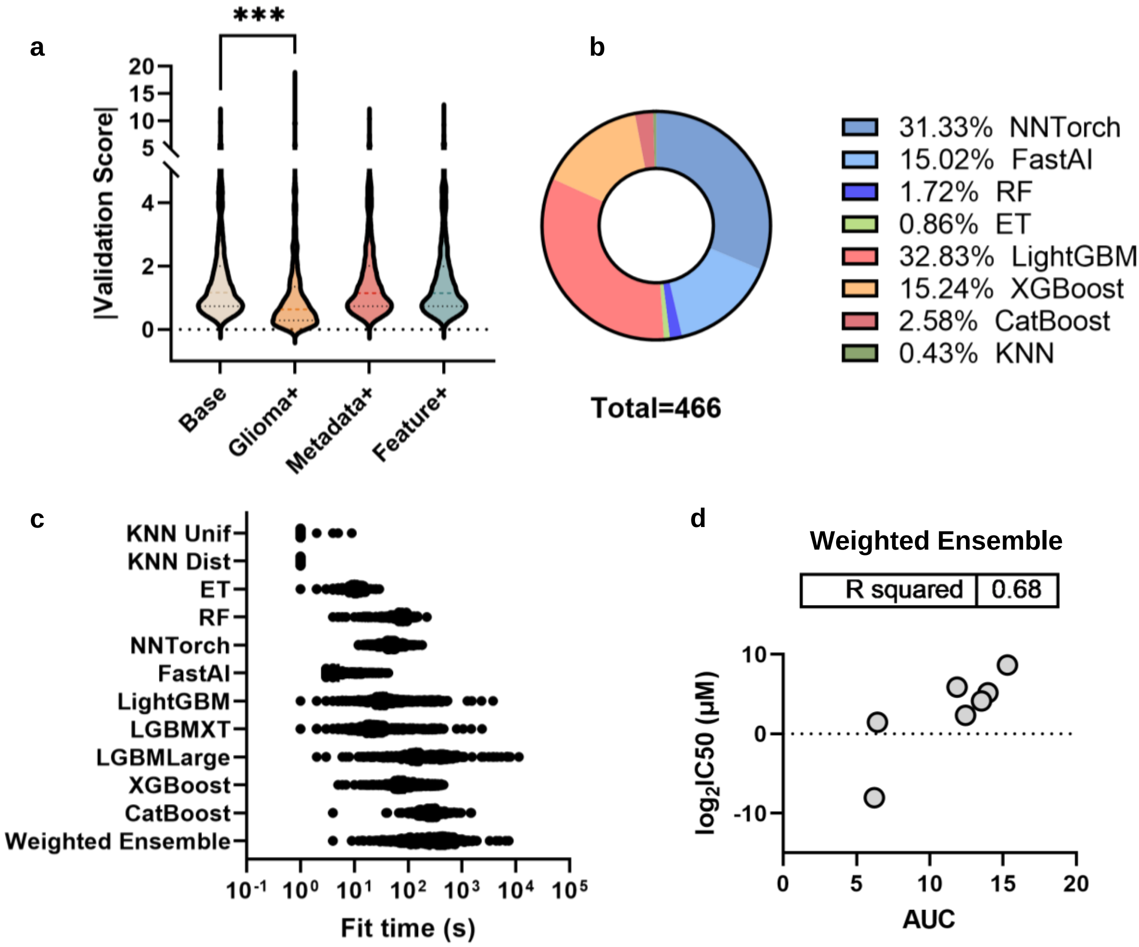

Supplementary Fig. S7. (a) Validation score of the GlioML base model and three types of enhancement models. Smaller absolute value indicates better performance after training. (b) The proportion of top-performing predictors generated by different single machine learning algorithms in the version of Glioma$^+$ enhanced model. (c) The fit-time of different single machine learning algorithms in the Glioma$^+$ enhanced model. (d) Linear regression analysis comparing Log-transformed IC_{50} values in GSC with predicted drug response AUC by the weighted ensemble model of Giloma$^+$ enhanced GlioML.

Median(%)	106	59	49	39	4.2	50	5.8
Mean(%)	105	60	63	39	11	50	7.5

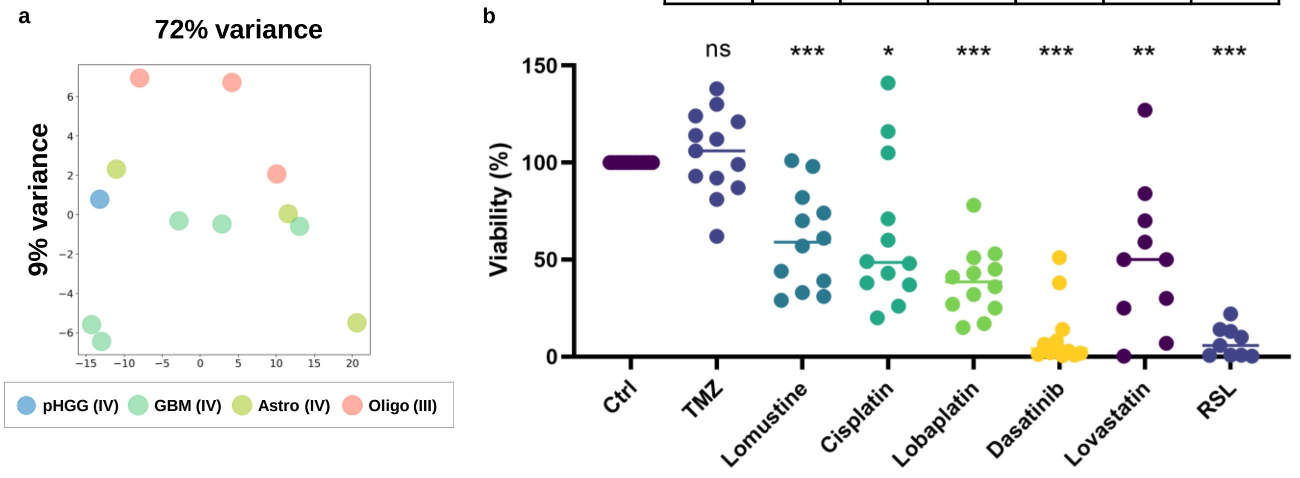

Supplementary Fig. S8. (a) Principal component analysis of GlioML-predicted drug fesponse patterns across 12 patient samples. (b) Comprehensive drug response plots for all patients covering clinical drugs and 3 additional GlioML-identified compounds.

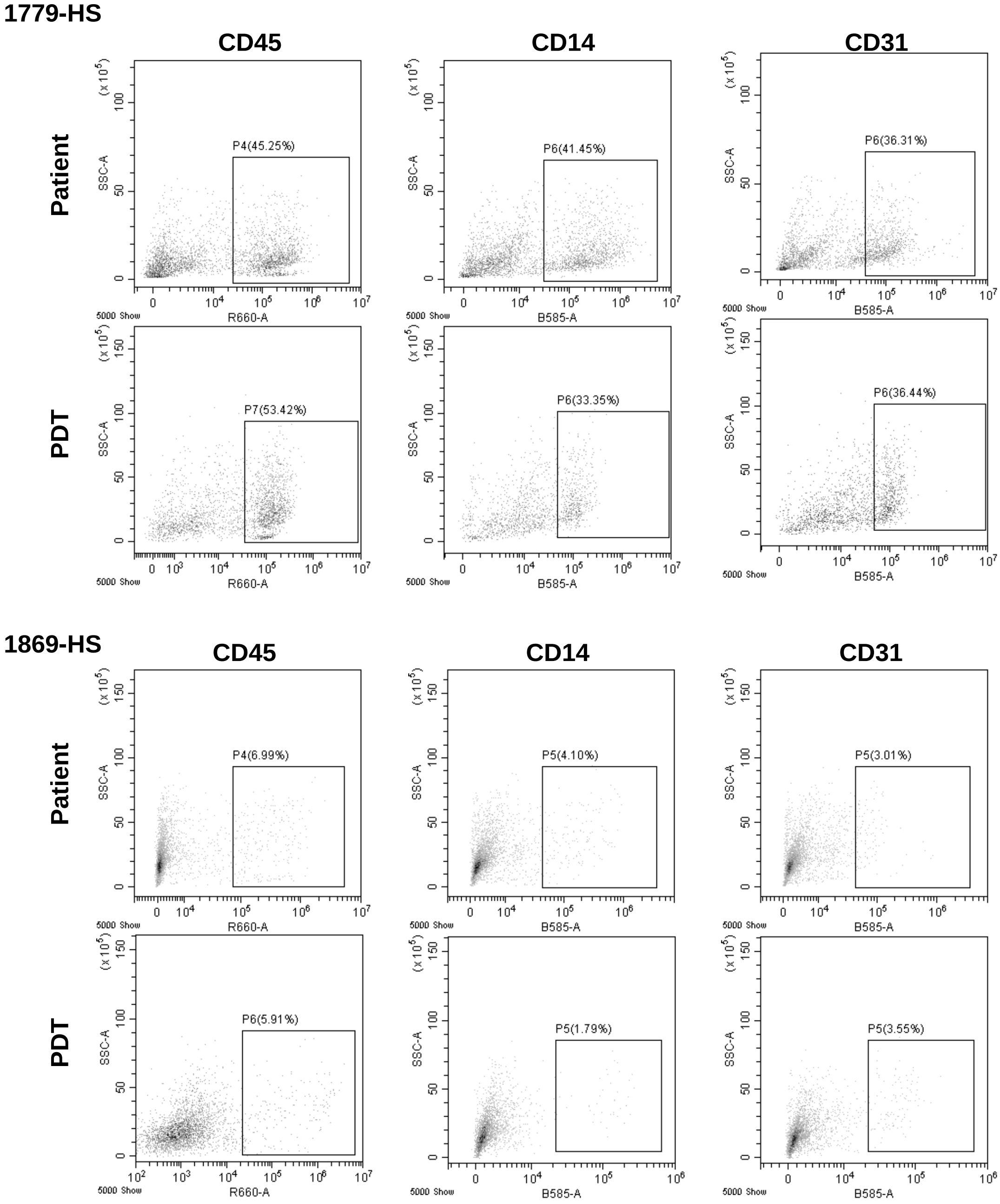

Supplementary Fig. S9. Flow cytometry analysis of two pairs of patient sample and corresponding PDT. CD45, CD14, and CD31 was stained and gated.

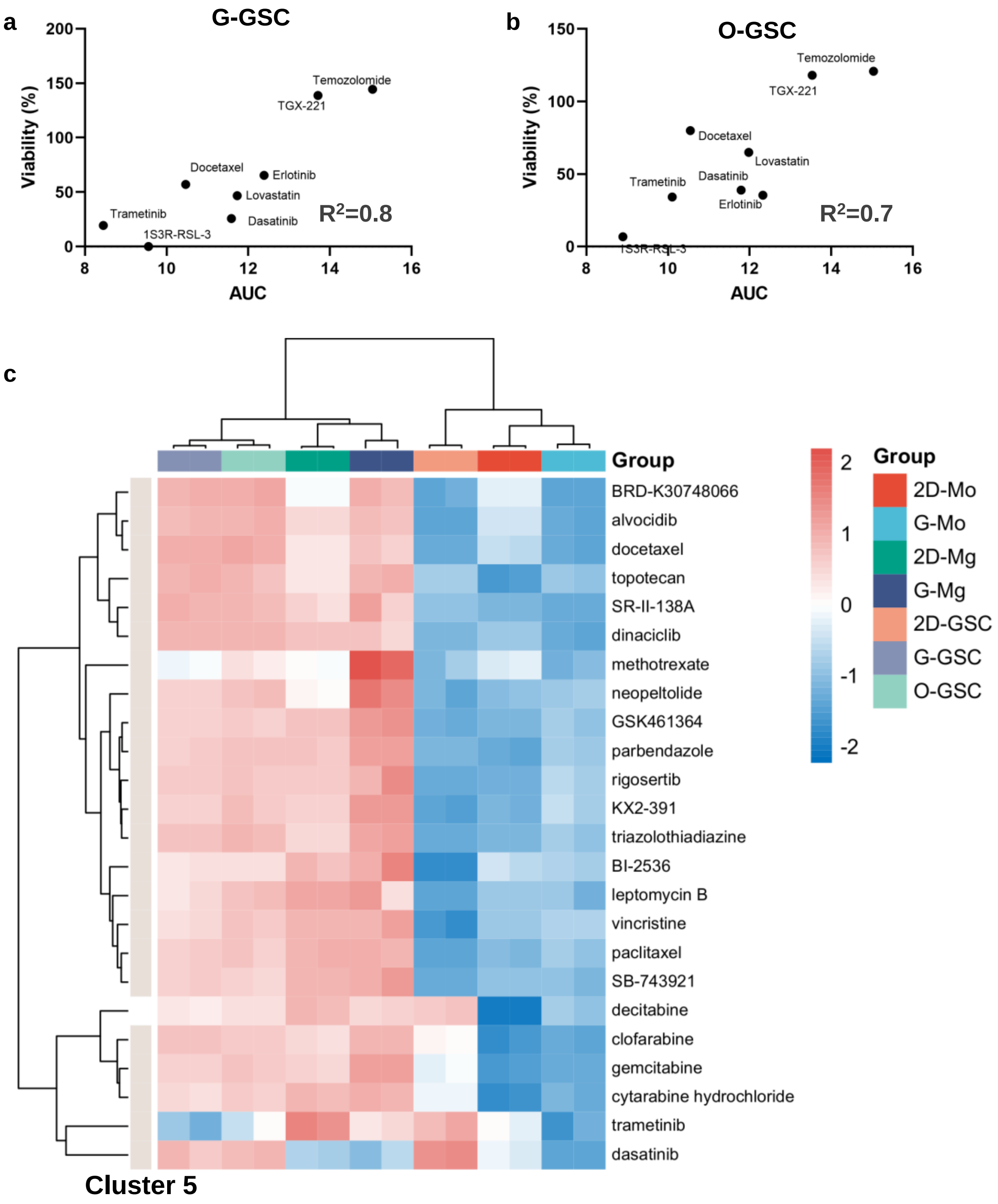

Supplementary Fig. S10. Simple linear regression of GlioML-predicted AUC and GSC viabilities in (a) GBM-Mg and (b) GBM-Mo models. (c) Heatmap of relative AUC values predicted by GlioML, showcasing the top 5% most differing compounds in two clusters. Traditional monocytes and GSC cultures, along with G-Mo, exhibited similar drug response patterns, whereas cocultured GSCs, G-Mg, and traditionally cultured microglia demonstrated similar response patterns.

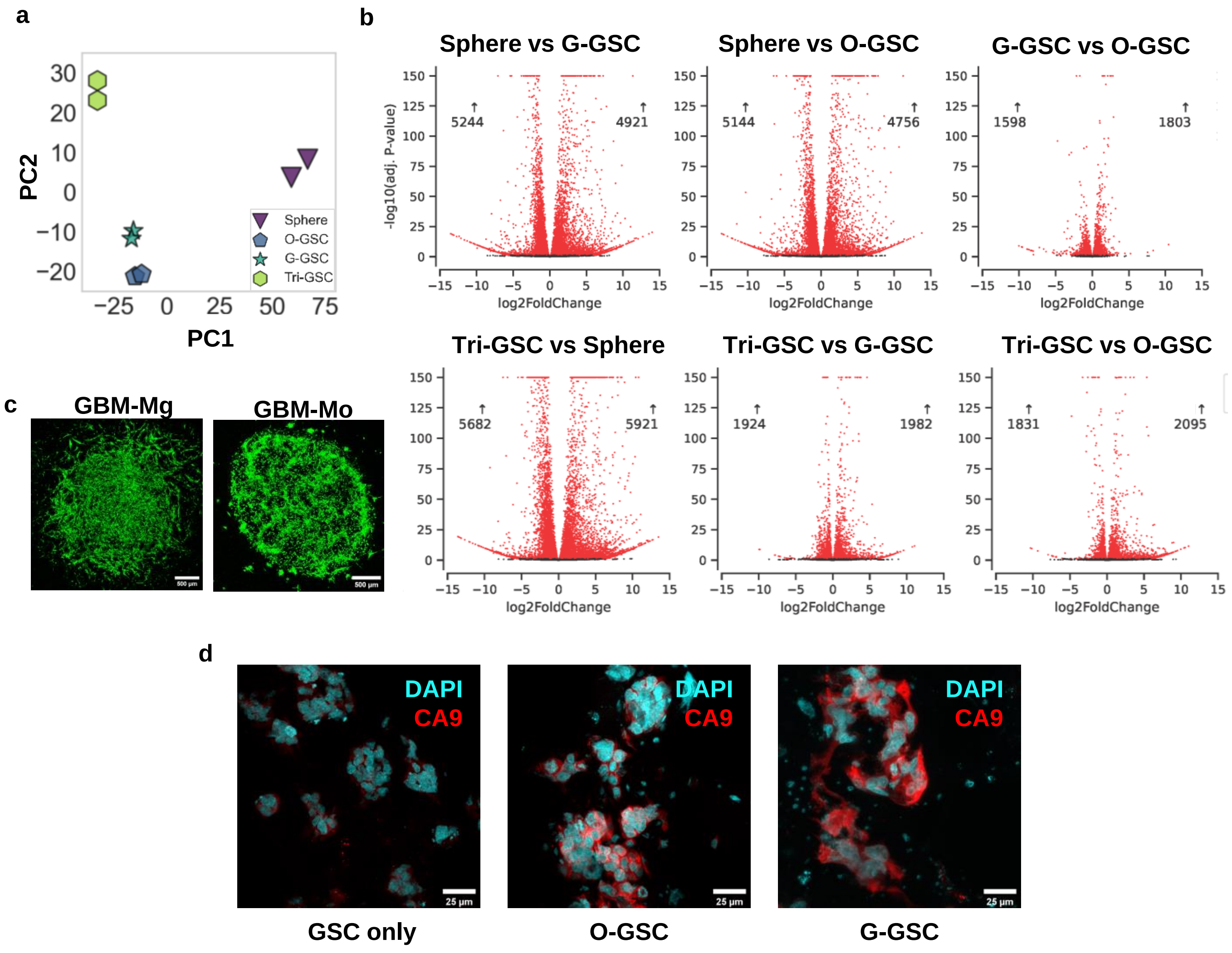

Supplementary Fig. S11. (a) Principal component analysis of gene expression in GSCs under various culture conditions. (b) Volcano plot comparison of GSCs in different culture types. (c) Fluorescent microscopic images of G-GSC and O-GSC in bioprinted co-culture models on day 7, with GSCs labeled with green fluorescent proteins. (d) Immunofluorescence staining of hypoxia marker CA9 with nuclei counterstained using DAPI.

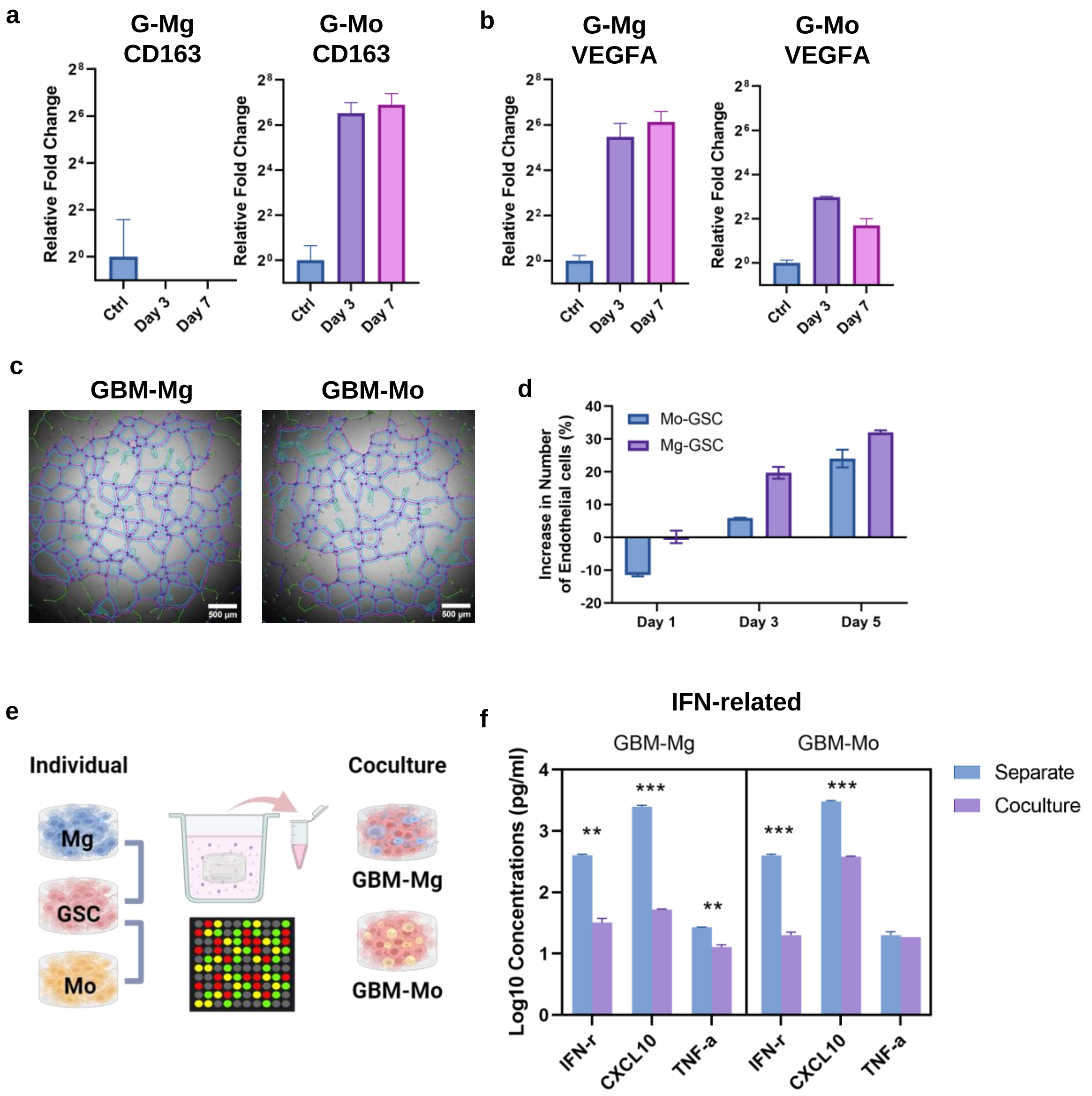

Supplementary Fig. S12. (a) RT-qPCR results showing relative fold change of CD163 gene expression in G-Mg and G-Mo at day 3 and day 7 isolated from bioprinted coculture compared to their 2D counterparts. (b) RT-qPCR results showing relative fold change of VEGFA gene expression in G-Mg and G-Mo at day 3 and day 7 isolated from bioprinted coculture compared to their 2D counterparts. (c) Endothelial cell tube formation on Matrigel cultured in GBM-Mg or GBM-Mo conditioned medium. (d) Assessment of endothelial cell growth in GBM-Mg or GBM-Mo conditioned medium. (e) Schematic illustration of cytokine profiling in bioprinted GBM-Mg, GBM-Mo, and single cell type models. (f) Absolute abundance of IFN-γ, CXCL10, and TNF-α in coculture supernatant compared to mixed supernatant from monocultures of involved cell types.

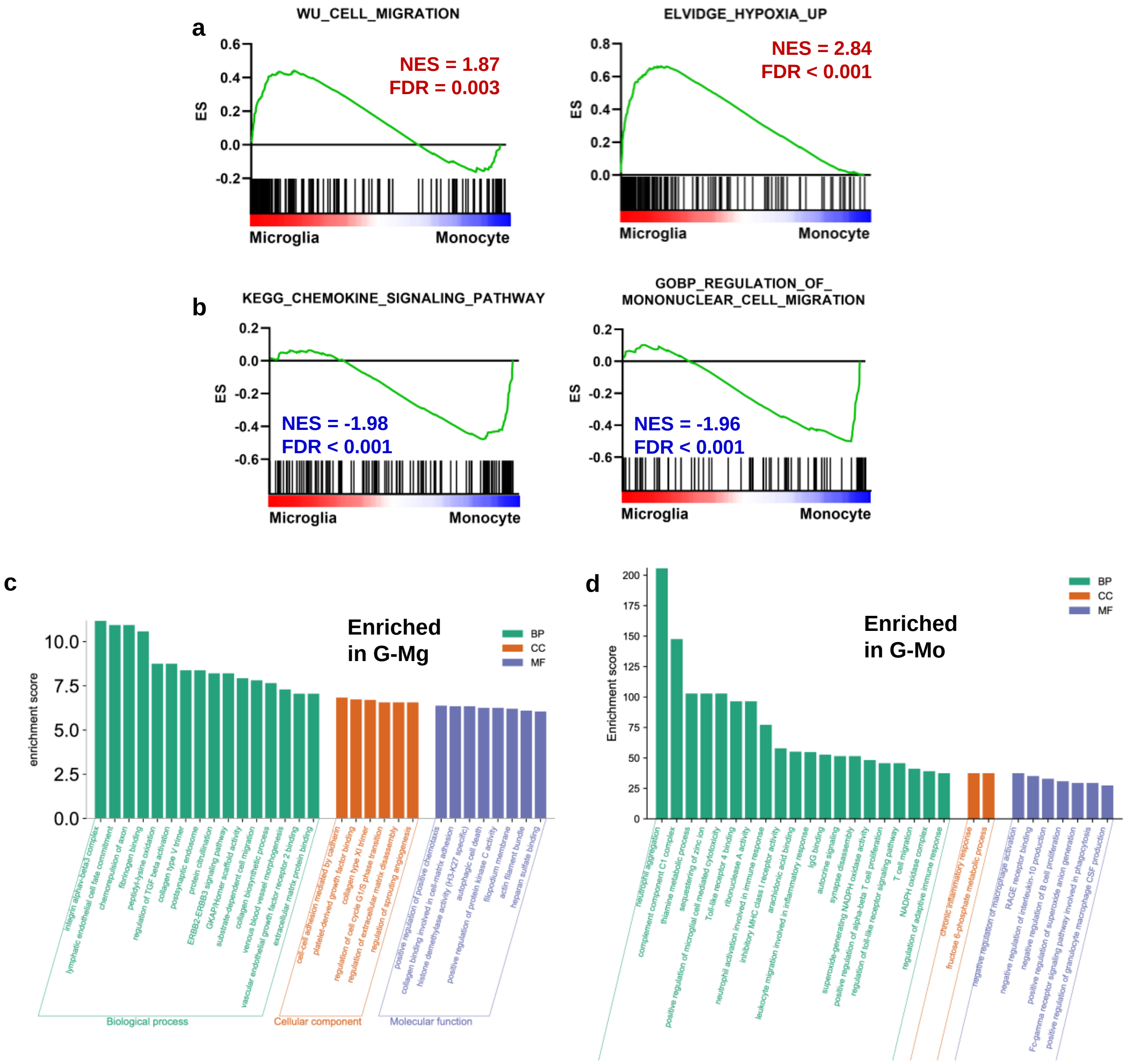

Supplementary Fig. S13. Gene set enrichment analysis showing that (a) hypoxia and cell migration pathways were significantly enriched in G-Mg compared to G-Mo, and (b) chemokine signaling and mononuclear cell migration pathways were significantly enriched in G-Mo compared to G-Mg. Gene ontology analysis showing enriched pathways in (c) G-Mg and (d) G-Mo.
